# Reduced Food Intake and Body Weight in Mice Deficient for the G Protein-Coupled Receptor GPR82

**DOI:** 10.1371/journal.pone.0029400

**Published:** 2011-12-28

**Authors:** Kathrin M. Y. Engel, Kristin Schröck, Daniel Teupser, Lesca Miriam Holdt, Anke Tönjes, Matthias Kern, Kerstin Dietrich, Peter Kovacs, Ute Krügel, Holger A. Scheidt, Jürgen Schiller, Daniel Huster, Gudrun A. Brockmann, Martin Augustin, Joachim Thiery, Matthias Blüher, Michael Stumvoll, Torsten Schöneberg, Angela Schulz

**Affiliations:** 1 Molecular Biochemistry, Institute of Biochemistry, Medical Faculty, University of Leipzig, Leipzig, Germany; 2 Institute of Laboratory Medicine, Clinical Chemistry and Molecular Diagnostics, Medical Faculty, University of Leipzig, Leipzig, Germany; 3 Department of Internal Medicine, Medical Faculty, University of Leipzig, Leipzig, Germany; 4 Interdisciplinary Centre for Clinical Research, Medical Faculty, University of Leipzig, Leipzig, Germany; 5 Rudolf Boehm Institute of Pharmacology and Toxicology, Medical Faculty, University of Leipzig, Leipzig, Germany; 6 Institute of Medical Physics and Biophysics, Medical Faculty, University of Leipzig, Leipzig, Germany; 7 Institute of Animal Sciences, Humboldt-Universität zu Berlin, Berlin, Germany; 8 Ingenium Pharmaceuticals AG, Martinsried, Germany; Sapienza University of Rome, Italy

## Abstract

G protein-coupled receptors (GPCR) are involved in the regulation of numerous physiological functions. Therefore, GPCR variants may have conferred important selective advantages during periods of human evolution. Indeed, several genomic loci with signatures of recent selection in humans contain GPCR genes among them the X-chromosomally located gene for GPR82. This gene encodes a so-called orphan GPCR with unknown function. To address the functional relevance of GPR82 gene-deficient mice were characterized. GPR82-deficient mice were viable, reproduced normally, and showed no gross anatomical abnormalities. However, GPR82-deficient mice have a reduced body weight and body fat content associated with a lower food intake. Moreover, GPR82-deficient mice showed decreased serum triacylglyceride levels, increased insulin sensitivity and glucose tolerance, most pronounced under Western diet. Because there were no differences in respiratory and metabolic rates between wild-type and GPR82-deficient mice our data suggest that GPR82 function influences food intake and, therefore, energy and body weight balance. GPR82 may represent a thrifty gene most probably representing an advantage during human expansion into new environments.

## Introduction

With the availability of large public data sets, the knowledge of many mammalian genomic sequences, and large-scale genotyping resources, numerous loci which have potentially been under selection in the entire human species, or locally in specific human populations have been identified [1], [2], [3], [4], [5], [6], [7]. G protein-coupled receptors (GPCR) represent the largest gene family in vertebrate genomes [8] and participate in the regulation of almost every physiological function. It is therefore very likely that GPCR variants provided selective advantage during periods of human evolution. Consistently, meta-analysis of genomic scans for signatures of selection revealed a number of such loci containing GPCR genes [9]. The X-chromosomal Ca^2+^/calmodulin-dependent serine protein kinase (CASK) locus which contains two orphan rhodopsin-like GPCR, GPR34 and GPR82, is one of these genomic regions and displays significant signatures of recent selection in human populations [6].

Both, GPR34 and GPR82 belong to the group of P2Y_12_-like receptors, all structurally related to the clopidogrel-sensitive ADP receptor P2Y_12_ [10], [11]. Other members of the P2Y_12_-like receptor group are the ADP receptor P2Y_13_, the UDP-glucose receptor P2Y_14_, and the orphan receptors GPR87 and GPR171 [12]. Recently, several members of the P2Y_12_-like receptor group have been assigned to nucleotide derivates and lipids as physiological ligands [13], [14], [15] among them GPR34 as receptor for *lyso*-phosphatidylserine (*lyso*-PS), a finding that is still under discussion [16]. GPR82 was first discovered by mining GenBank for novel GPCR sequences [17]. However, the link between both, the potential selection of the genomic locus containing GPR34 and GPR82 and the physiological relevance of the two GPCR is still unknown.

Herein, we provide further evidence of recent selection at the human X-chromosomal CASK locus. Fine-mapping of this locus by sequencing a 10-kbp region in two human populations supported this finding. Because the functional relevance of CASK and GPR34 was studied elsewhere [18], [19] we addressed the physiological relevance of the orphan receptor GPR82 by characterizing a GPR82-deficient (KO) mouse strain. KO mice show a distinct phenotype with reduced body weight, body fat content and food intake compared to their wild-type (WT) littermates. Further, in GPR82-deficient mice the triacylglyceride (TAG) levels in plasma and in LDL and VLDL lipoprotein fractions are reduced and glucose tolerance is increased. Our data indicate a role of GPR82 in the regulation of energy homoeostasis in mice suggesting this orphan GPCR as a candidate gene for optimal energy accumulation during human evolution.

## Results

### The CASK locus shows signatures of recent selection

The X-chromosomal CASK locus, which contains the two orphan GPCR, GPR34 and GPR82, attracted our attention because it showed high significance in iHS (Integrated Haplotype Score) analysis [6]. To verify these initial findings SNP data of the CASK locus from European, Asian and African HapMap individuals and a data set of a local European population (Sorbs [20]) were analyzed with additional statistical methods (Tajimas D, Fu/Li D and F tests). In contrast to European and African samples, Asian individuals showed no SNP variations (monomorph) indicating a long, recently fixed haplotype. To properly analyze the monomorphic Asian (HCB) data set, the ancestral sequence was used for contrasting purposes. As presented in Table 1, the SNP analyses of the HapMap European (CEU) and Chinese (HCB) data set revealed significant signatures of selection in more than one test.

**Table 1:** Search for signatures of recent selection in human populations.

*Interval size (bp)*	*Population*	*# Sequences*	*# Sites*	*Tajima's D*	*Fu and Li's D*	*Fu and Li's F*
**100 k**	**CEU**	90	146	1.37	1.95*	2.06*
	**HCB**	68	144	1.08	1.34	1.48
	**HCB+ancestral**	69	144	−1.77	−5.09*	−4.58*
	**YRI**	90	143	−0.29	2.17*	1.39
	**Sorbs 500 k**	679	53	3.18**	1.79*	2.97*
**10 k**	**Sub-Saharan Africans** #	27	9961	−0.95	0.04	−0.32
	**Asians** #	23	9969	−2.20**	−3.38*	−3.53*

SNP data (approx. 100 kbp up- and downstream of the GPR34/GPR82 genomic position) from HapMap database (Phase II) [35] individuals (CEU: European, HCB: Chinese, YRI: African) and a local Sorbs cohort [20], retrieved from a 500 K Affymetrix GeneChip were analyzed for signatures of recent selection (for details see *Materials and Methods*). Because the SNP data of HCB were almost monomorph, the ancestral sequence was defined by comparison with the respective sequence of the chimpanzee and included in the analyses.

# A 10-kbp genomic fragment of the GPR34/GPR82 genomic region was amplified from 50 male individuals (23 Asians, 27 African individuals) (for details see *Materials and Methods*) and analyzed for signatures of recent selection.

*P<0.02;

**P<0.01.

All methods applied above (Tajimas D, Fu/Li D and F) test for reduction of genetic diversity and excess of rare alleles [5]. Although these tests can be performed with SNP data, biased SNP assignments may mask genetic diversity in some populations (e.g. in Asians). Therefore, we sequenced a 10-kbp genomic fragment in Asian and Sub-Saharan African individuals (only hemizygous male individuals to doubtlessly assign haplotypes). Again, Asian individuals were almost monomorph whereas African individuals displayed several haplotypes. Analyses confirmed highly significant D and F values all consistent with recent selection of the CASK locus in the Asian population (Table 1). Interestingly, the haplotypes were identical in individuals from distant geographic regions (Beijing/China, Korea, Vietnam).

Taken together, our data are compatible with the hypothesis that the CASK locus underwent recent selection in human populations. This locus contains three genes, CASK, GPR34 and GPR82. Because the functional relevance of CASK and GPR34 has recently been evaluated (see *Discussion*) but not the one of GPR82, we studied this orphan GPCR in more detail.

### GPR82-deficient mice are lean, have decreased triacylglyceride plasma levels and improved glucose tolerance

GPR82 is structurally related to P2Y_12_-like receptors and became relevant in the very early vertebrate evolution. Although present in most vertebrate genomes GPR82 function is not required for unknown reasons in some fish (puffer fish), reptile (anole) and mammalian (otolemur) species (details on GPR82 evolution are provided on request). GPR82 is ubiquitously expressed with highest mRNA levels in epididymis, testis and hypothalamus (*Table S1*). GPR82 shows conserved transcript and promoter structures indicating that the GPR82 gene is functional in mouse and human (*Figure S1*). To date, the endogenous agonist is unknown and all our attempts to determine the G protein-coupling abilities failed so far (data not shown).

To gain insight into the functional relevance of GPR82, a GPR82-deficient mouse line was established by targeted gene deletion (*Methods S1*, *Figure S2*). The mice background was C3HeB/FeJ and animals of generations >12 were used for the study. Because the GPR82 gene lies within an intron of the CASK gene and the targeting construct may affect CASK expression CASK mRNA levels were quantified in different tissues of WT and KO. As shown in *Figure S2C* there were no significant differences in mRNA expression levels of CASK.

Because of the X-chromosomal localization of GPR82 heterozygous females were mated with WT and KO males to produce all possible genotypes of both sexes. Offspring of all genotypes were vital and fertile. Although the genotype distribution was as expected when KO males were mated with heterozygous females, there was a preference of the KO allele in WT male/heterozygous female matings (*Figure S3*). The reason remains unclear.

**Figure 1:**
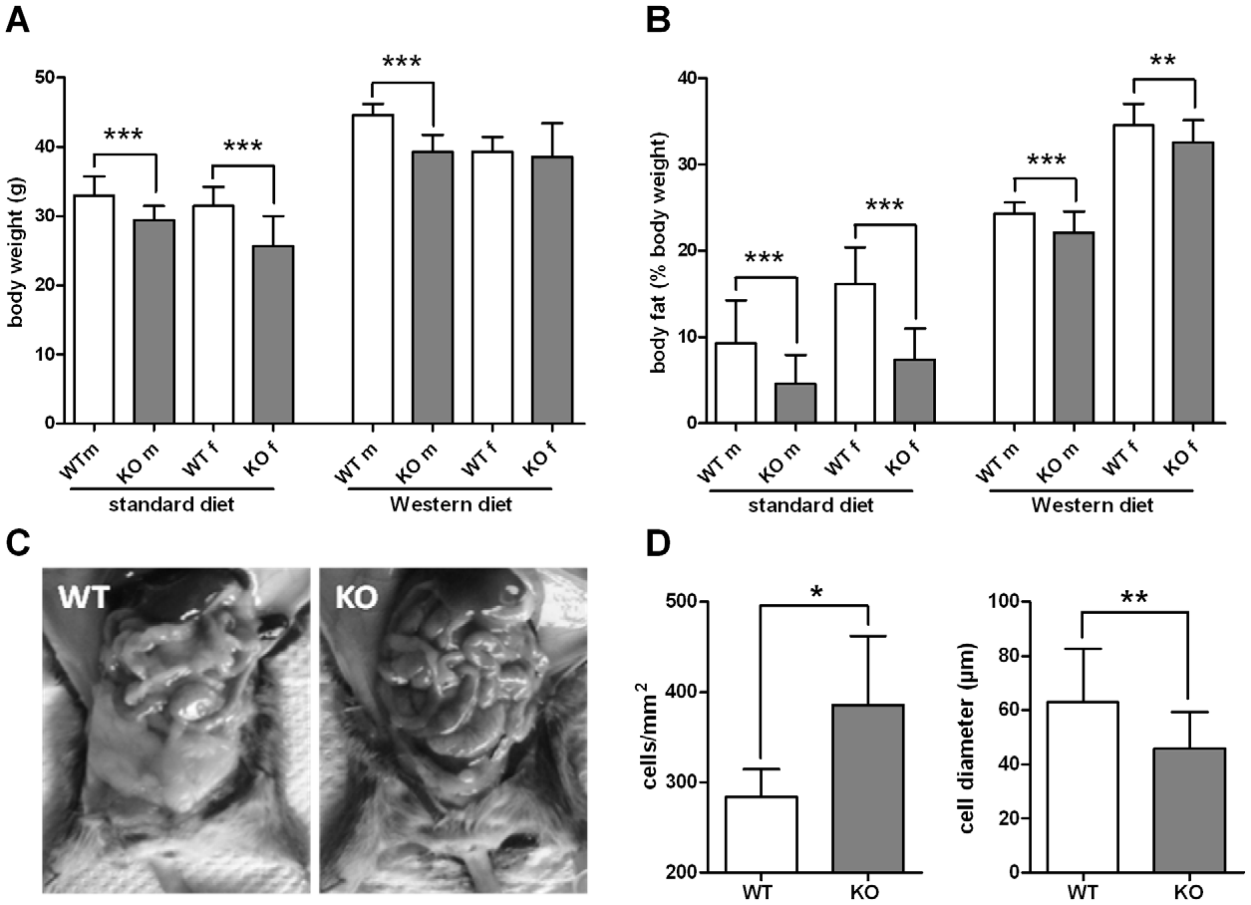
Body weight and fat content of WT and GPR82-KO mice. (**A**) Body weight in 15-week-old mice under standard diet and Western diet. Body weights of animals under standard diet (male: WT *n* = 26, KO *n* = 30; female: WT *n* = 10, KO *n* = 7) showed a significant difference between WT (open bars) and KO mice (filled bars). Animals under Western diet (male: WT *n* = 26, KO *n* = 28; female: WT *n* = 24, KO *n* = 22) developed higher body weights and the weight differences between male WT and KO mice remained significant. (**B**). Body fat content of mice under standard (male: WT *n* = 26, KO *n* = 30; female: WT *n* = 10, KO *n* = 7) and Western diet (male: WT/KO *n* = 10; female: WT/KO *n* = 9) determined by QMR. The body fat content of KO mice was significantly reduced compared to WT animals under standard and Western diet. (**C**) Differences in visceral fat content between 3-month-old female WT and KO mice were already macroscopically found under standard diet. (**D**) The average size of adipocytes in perigonadal fat of male KO mice was smaller than in WT kept under Western diet and, therefore, the adipocyte number per mm^2^ was higher in KO. Data are given as mean ± SD. The body weight development of WT and KO mice under standard and Western diet from 4 to 14 weeks is given in *Figure S5*. *P<0.05, **P<0.01, ***P<0.001.

Because GPR82 is widely expressed, 3-month-old mice were subjected to a hypothesis-free phenotypic screen. This included morphological and histological measures, behavioral tests and hematological and chemical assessment of blood samples (summarized in Table 2). Most obvious KO mice developed a lower body weight compared to their WT littermates (Figure 1A, *Figure S5*). To further investigate this difference, 3-month-old animals were dissected and organs and tissues were morphologically and histologically analyzed. Organs showed no gross histological differences and no or a rather small reduction in the absolute weight (*Table S2*) suggesting that tissues not included in the weight analysis, such as adipose tissue, muscles and bones, account for the differences. Quantitative magnetic resonance (QMR) analyses revealed that KO mice of both genders have a significant lower absolute and relative body fat content compared to WT littermates (Figure 1B, *Table S5*). The lean mass relative to the body weight was significantly higher in KO than in WT mice (*Table S5*). A reduced visceral fat mass in KO animals was obvious during dissection of peritoneal organs (Figure 1C). As determined from histological sections [21], the average size of adipocytes in perigonadal fat pads was significantly lower (Figure 1D). Further, increased hepatic leptin receptor expression in GPR82-deficient mice (*Table S9*) indirectly reflects a lower body fat mass [22].

**Table 2:** Basic characterization of GPR82-KO mice.

*Parameter*	*Result*
***Morphology and Histology***	
body length, tail length, fur development, ear erection, abnormalities in teeth or extremities	no abnormalities detected
weight development, weight of organs	body weight of KO is significantly lower; some significant differences in relative organ weights (*Table S2*)
eosin/hematoxylin staining of histological slices of main organs	smaller adipocytes in KO (Figure 1D), no further abnormalities detected
***Clinical laboratory examinations***	
serum parameters (metabolites, enzymes), blood glucose, urine osmolality, differential hemogram, AA/AC-Screening	lower triglyceride levels in KO, for further significant differences see *Tables S3*, *S4*, *S5*, *S6*
***Behavioral assays***	
SHIRPA protocol	see. *Table S7*
hot plate test	increased heat tolerance of KO (*Figure S4*)
open field test	significant lower velocity and counts in KO (*Table S7*)
light-dark test	no significant differences
***Immunological challenging***	
DTH test	significant decrease in paw swelling in KO mice (*Table S8*)

An initial screen was performed to characterize GPR82 deficiency in mice kept under SPF conditions. Detailed experimental setups and results are given in Methods S1.

**Figure 2:**
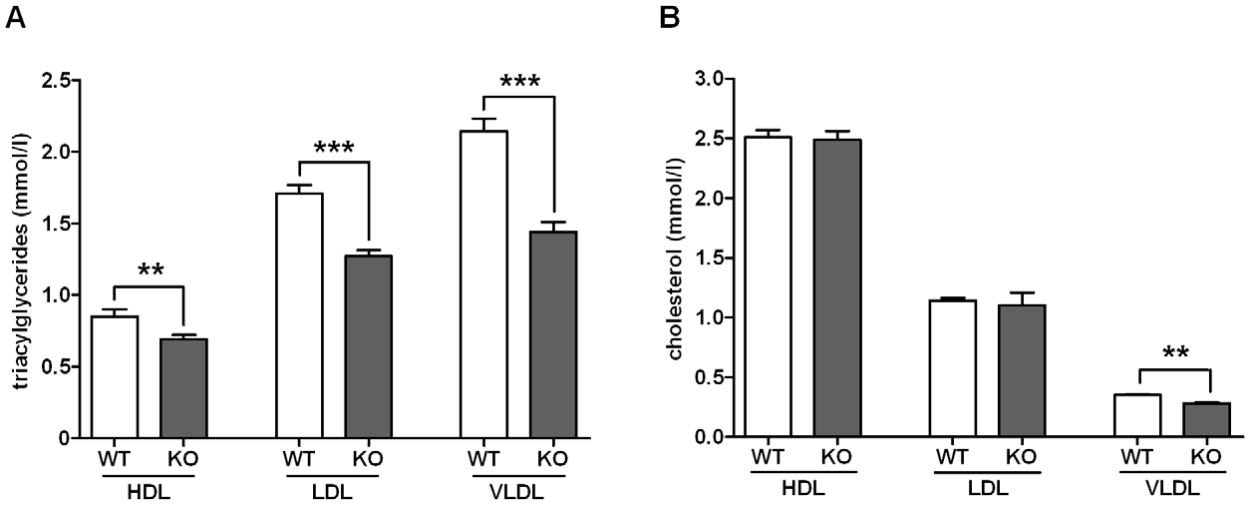
Serum triacylglyceride (TAG) and cholesterol levels of WT and GPR82-KO mice. Concentration of TAG and cholesterol in lipid fractions (see *Methods S1*) of male WT (open bars, *n* = 39) and male KO animals (filled bars, *n* = 45). Lipid fractions were obtained by ultracentrifugation. (**A**) TAG of KO mice were significantly lower in HDL, LDL and VLDL fractions, whereas (**B**) cholesterol concentrations were different between KO and WT animals only in VLDL. Female animals gave essentially similar results (see *Table S5*). Concentrations are given as mean ± SEM. **P<0.01, ***P<0.001.

Except for significantly lower TAG levels in KO mice, the hematological and chemical assessment of blood samples including concentration determination of acylcarnitines as metabolites of free fatty acids revealed no major differences between WT and KO mice (see *Tables S3*, *S4*, *S5*). Detailed analyses of TAG and cholesterol levels in plasma lipoproteins indicated that TAG levels were significantly lower in all three lipoprotein fractions (LDL, HDL, VLDL) of KO mice (Figure 2A), whereas cholesterol was found reduced only in VLDL (Figure 2B).

**Figure 3:**
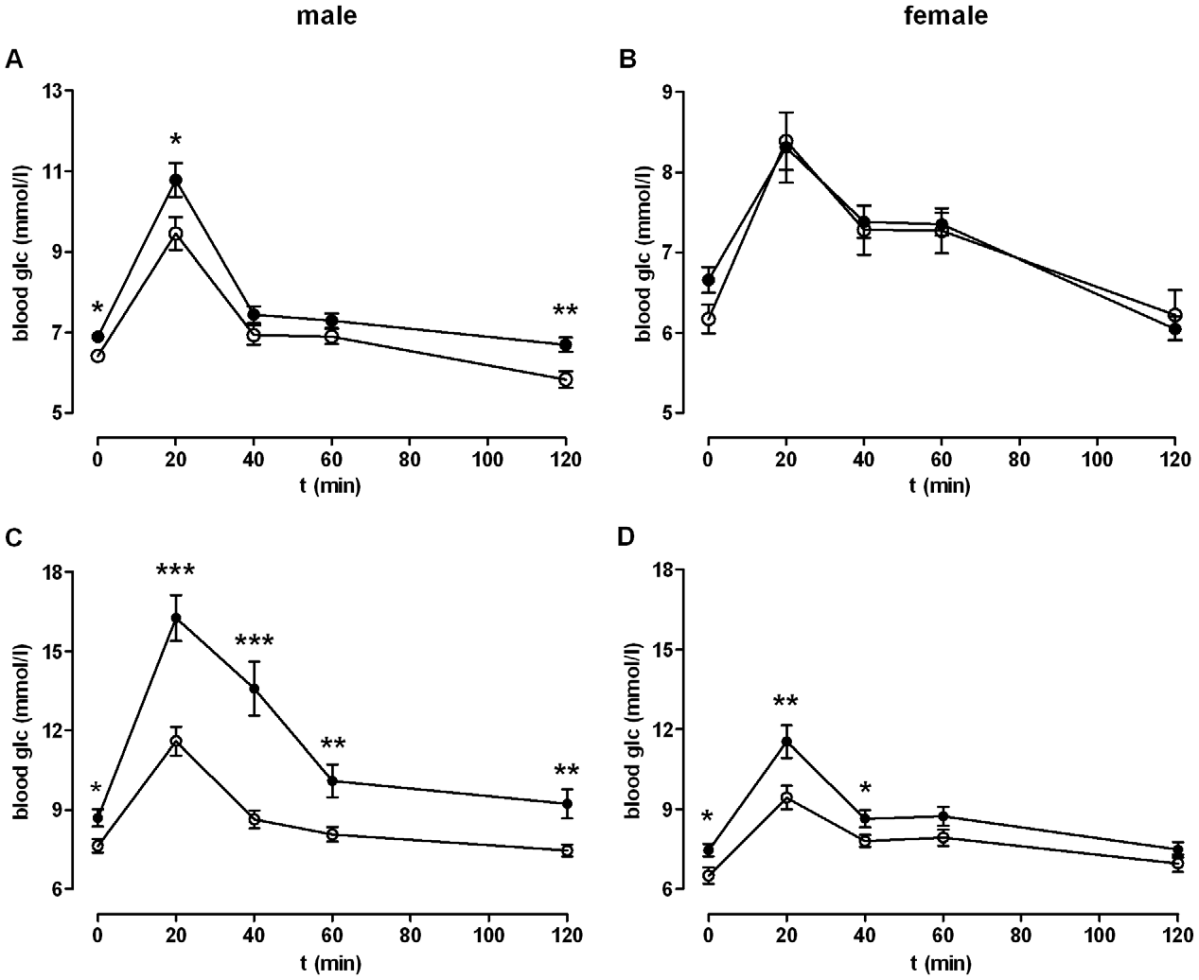
Oral glucose tolerance test (oGTT). An oGTT was performed after a starving period over night by oral application of 80 mg glucose. WT data are given as black dots, KO data as open dots. Blood glucose was measured before (given as 0 min) and 20, 40, 60 and 120 min after glucose application. (**A**) In male KO animals (*n* = 22) the blood glucose at 0 min was significantly lower compared to WT male mice (*n* = 25). The glucose levels at 20 and 120 min after glucose application increased in KO male mice but were also significantly lower than in WT male mice. (**B**) Female KO mice (*n* = 10) did not show any differences in their blood glucose levels compared to WT females (*n* = 17). OGTT was also performed with (**C**) male (WT/KO *n* = 19/15) and (**D**) female mice (WT/KO *n* = 21/14) after a 12 week Western diet. Results are given as mean ± SEM. *P<0.05, **P<0.01, ***P<0.001.

To address possible consequences of the reduced body weight in KO mice oral glucose tolerance tests (oGTT) were performed. Fasting blood glucose levels were lower in male KO mice and KO animals showed a significantly improved glucose tolerance (Figure 3A, B). Fasting serum insulin levels were only slightly lower in KO (*Table S5*) and hepatic insulin receptor expression was not different (*Table S9*).

### Challenging the Phenotype by a Western Diet

To test whether the phenotypical differences in lipid and glucose metabolism can be challenged by diet, one-month-old mice were kept under Western diet *ad libitum* for further 3 months. The body weight of both, WT and KO mice, was higher under Western diet, however, the difference in body weight between both genotypes remained at 16% (Figure 1A). Similarly, the difference in the body fat content measured by QMR remained unchanged compared to animals under standard diet (Figure 1B). As expected, glucose tolerance of WT mice was reduced under the Western diet. Although the glucose tolerance was also reduced in KO animals the contribution of the Western diet was not as pronounced as in WT animals (Figure 3C/D, *Table S6*). The insulin concentration in serum of starved animals was significantly lower in KO animals on Western diet (*Table S6*). This is consistent with the other symptoms of a metabolic syndrome found in WT mice kept under Western diet.

**Figure 4:**
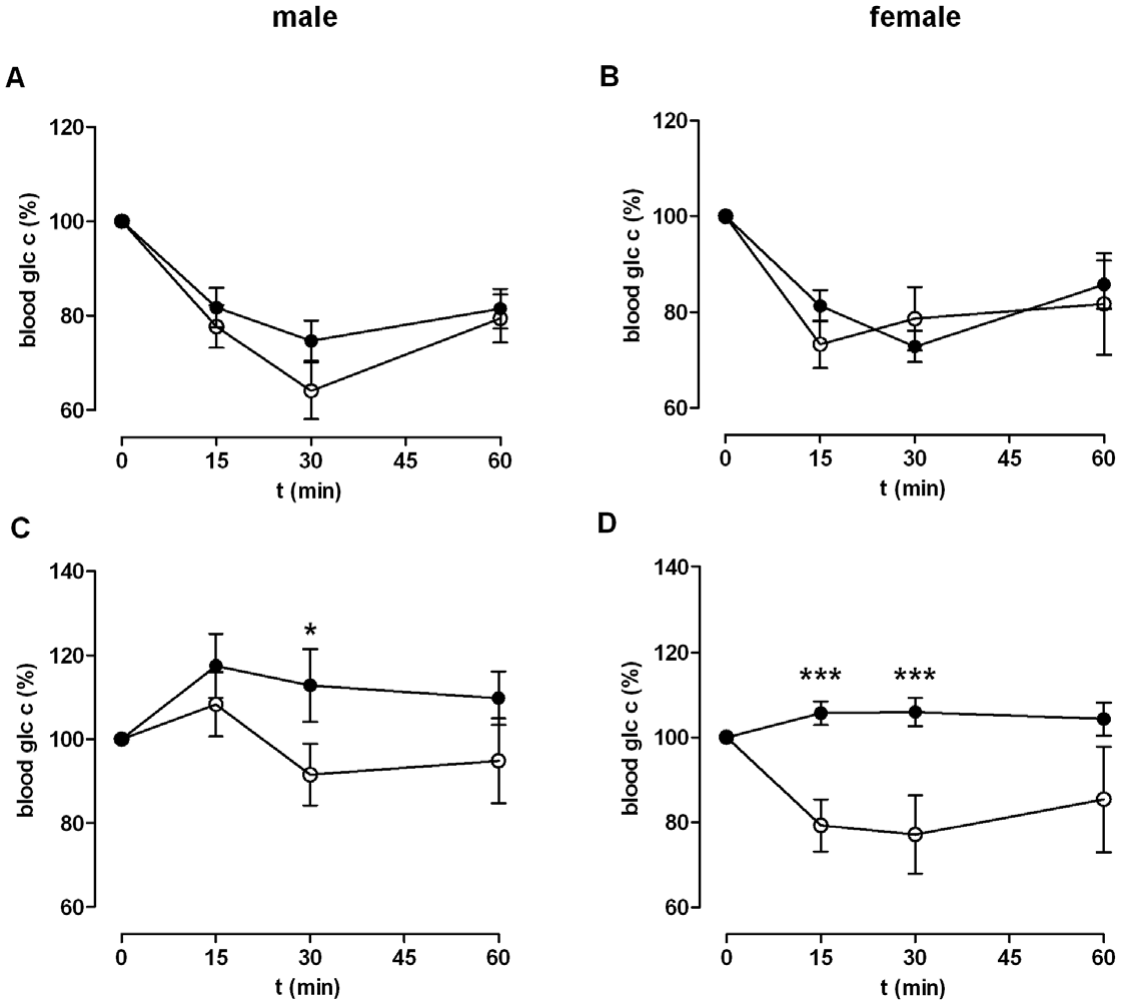
Insulin tolerance test (ITT). An ITT was performed by i.p. application of 0.75 U insulin/kg body weight. WT data are given as black dots, KO data as open dots. Blood glucose was measured before (given as 0 min) and 15, 30, and 60 min after insulin application. (**A**) In male KO animals (*n* = 7, blood glucose_0 min_ 7.01±0.22 mmol/l) the change of blood glucose concentration did not show any differences compared to male WT (*n* = 11, 6.75±0.52 mmol/l). (**B**) Similar, female KO (*n* = 12, 6.26±0.29 mmol/l) did not show any differences in their blood glucose levels compared to female WT (*n* = 12, 5.38±0.32 mmol/l). ITT was also performed with (**C**) male mice (WT/KO, *n* = 13/12, 8.33±0.74/5.57±0.36 mmol/l) and (**D**) female (WT/KO, *n* = 15/13, 6.75±0.21/6.45±0.25 mmol/l) after a 12 week Western diet. Changes in blood glucose concentration are given as mean ± SEM. *P<0.05, **P<0.01, ***P<0.001.

To answer the question whether the enteral glucose uptake is disturbed in KO mice, serum glucose levels over time after oral and intraperitoneal (i.p.) application were compared. As shown in *Figure S6*, an improved glucose tolerance in KO mice was seen in Western diet-fed mice following i.p. glucose application. This indicates that a reduced enteral glucose uptake is not responsible for lower blood glucose levels in oGTT in KO mice. Next, insulin tolerance was tested by an i.p. insulin tolerance test (ITT). No significant changes in insulin-induced reduction of plasma glucose levels were observed between WT and KO mice under standard diet (Figure 4A, B). However, male WT mice fed with Western diet showed significantly higher basal glucose levels compared to male KO mice and almost no changes in glucose levels after insulin application (Figure 4C). Female KO mice under Western diet, showed significantly improved insulin sensitivity compared to WT controls (Figure 4D). This indicates that diet-induced insulin resistance is significantly reduced in female GPR82-deficient mice.

### Molecular characterization of adipose tissue in WT and KO mice

To screen for differences in the lipid composition of different adipose tissues in KO and WT ^1^H high-resolution magic angle spinning (HRMAS) NMR and MALDI-TOF mass spectrometry were performed. The ^1^H HRMAS spectra provided well separated peaks for the methyl and methylene segments from the TAG lipid chains of the fat tissue as well as a water resonance (*Figures S7A, B*). As NMR is a quantitative method, the peak integral ratios CH_2_/water and CH_3_/water (called “normalized peak integral”) provide a direct measure of the fat content of the respective tissue. Under standard diet no significant differences were observed between WT and KO mice in perigonadal, subcutaneous, and brown adipose tissue (*Figure S7C*). However, fat tissues from Western diet-fed mice showed a diet-induced increase in the fat-to-water ratio, e.g. an increase in the fat content with significant differences in the subcutaneous fat tissue between WT and KO (*Figure S7C*).

A more detailed analysis of the composition of perigonadal, subcutaneous and brown fat tissue was performed by mass spectrometry. Specifically, the relative amounts of TAG were determined as previously described [23]. As shown in *Figure S8*, there are only minor differences in the TAG composition between WT and KO mice. However, the Western diet had a significant effect on the overall TAG composition in all three fat tissues. Specifically, lower molecular weight TAG, i.e. TAG with shorter fatty acyl residues and oxidized TAG species with shortened fatty acyl residues were more abundant in Western diet-fed mice. Because there were no significant differences in the TAG pattern between WT and KO mice, we interpreted the differences obtained by NMR under Western diet as an increase in the relative amount of fat compared to water in the fat tissues of WT mice.

### Food intake is reduced in GPR82-deficient mice

**Figure 5:**
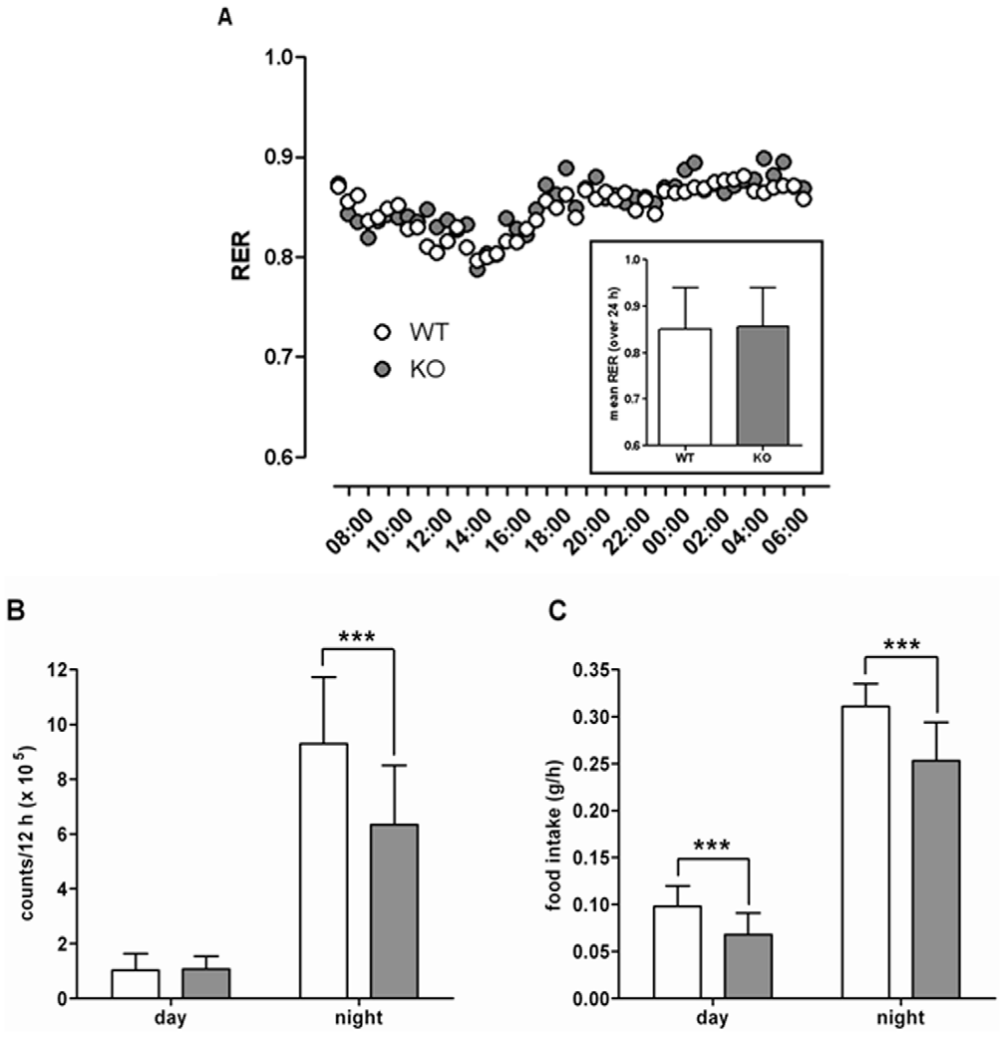
Respiratory rate, circadian motor activity and food intake. (**A**) 20-week-old male mice (9 WT and 8 KO) were adapted to metabolic cages (TSE Systems, Bad Homburg, Germany) and monitored over 24 h. Further results of the metabolic cage analysis are given in *Table S10*. The respiratory rates are given as mean, the inset represents the means ± SD over 24 h. (**B**) The motor activity of WT (open bars) and KO (filled bars) mice was monitored over 3 weeks in a TSE InfraMot (TSE-Systems). The sensors register the activity of the subjects by sensing the body-heat image, i.e. infrared radiation, and its spatial displacement over time. Recorded data were pooled over the light (day) and dark (night) cycles. Because there was no significant difference between the beginning and end of the experiments, data of 3 weeks were pooled. Data of two independent experiments (*n* = 5 per experiment and genotype, male mice) are given as mean ± SD. (**C**) Food intake of WT (open bars) and KO (filled bars) mice was monitored over 3 weeks. Data of two independent experiments (*n* = 6 per experiment and genotype, male mice) are given as mean ± SD. ***P<0.001.

To test the hypothesis that reduced body fat is caused by an increased energy utilization or reduced food intake, respiratory and metabolic rates, body temperature, motor activity, and food intake were continuously monitored over 24 hours (temperature, metabolic cage) and 3 weeks (activity, food intake). KO mice showed a small but significant increase in body temperature (approximately 0.3°C) (*Figure S9*). Next, the energy balance was determined in metabolic cages. There were no differences in the respiratory (Figure 5A), and metabolic (*Table S10*) rates almost excluding temperature-driven effects on body weight. KO mice showed a reduced motor activity (*Table S10*) that was further analyzed over a 3 week period. The motor activity was significantly lower in KO animals during the dark period but not during light phase (Figure 5B). Reduced locomotion was also seen in the open field test (*Table S7*). In parallel, food intake was determined and KO mice showed a significant reduction during the light and the dark phase (Figure 5C).

Since GPR82 is highly expressed in the hypothalamus (*Table S1*), one can speculate that this GPCR is also involved in hypothalamic appetite regulation. As studied by qPCR, different diets had no effect on GPR82 expression (data not shown) and genes known to be involved in hypothalamic regulation of food intake showed no significant changes in expression levels (*Table S9*).

### Genotype-phenotype association in humans

The findings with GPR82-deficient mice encouraged us to screen for SNP-phenotype correlations with specific focus on BMI, fat content, serum glucose, and TAG in humans. We performed phenotype-genotype association studies in a Sorbs cohort that has been extensively phenotyped previously including metabolic and clinical parameters relevant for our purposes [20]. As with other populations, we found strong signatures of recent selection at the CASK locus in the Sorbs cohort (Table 1). More than 900 individuals of this cohort were genotyped at seven tagging-SNP sites spanning about 55 kb covering the genes of GPR34 and GPR82 (*Figure S10*). BMI and body fat content of males showed the most significant association with two tagging SNP, rs6609159 and rs4827286, in close 5′ and 3′ proximity to the GPR82 gene, respectively and for all phenotypes tested (*Table S11*). In males, SNP rs660915 and rs4827286 explained BMI differences of 0.7 and 0.74, respectively. Alleles associated with lower BMI values were linked to alleles associated with lower body fat mass. These associations were not found in female individuals. A tendency of gender specificity was also found in mice where male animals displayed more pronounced phenotypes specifically under Western diet (see Figure 3, *Figure S5*, *Table S6*). Interestingly, the SNP variants associated with lower BMI and body fat content have a frequency of approximately 35% in the Sorbs and are close to fixation in the Asian population (about 85%, see HapMap data). Significant correlations of two tagging SNP with the 120-min blood glucose level in oGTT were also found in female individuals when corrected for body fat content (data not shown). This indicates that the genomic CASK region also plays a role in glucose tolerance in humans. Most other parameters including those found to be significantly changed in KO mice displayed no significant association. Meta-analysis of the genotype-BMI correlations including two additional independent cohorts (Leipzig, ∼530 healthy individuals, [24] and Leipzig Heart Study, ∼1,900 individuals [25]) supported significant associations initially observed in the Sorbs cohort (*Table S12*).

## Discussion

The X-chromosomal CASK locus is characterized by long haplotypes, a reduced genetic diversity and an excess of rare alleles in some human populations (Table 1). This is compatible with the hypothesis that this locus underwent recent selection in human populations. The genomic locus contains three assigned genes: CASK, GPR34 and GPR82. To provide a link between selected and non-selected alleles, their phenotypic consequences and the selective force that may have produced the footprint of selection, information about the physiological relevance of these three genes is required.

CASK is an evolutionarily conserved multidomain protein composed of an N-terminal Ca^2+^/calmodulin-kinase domain, central PDZ and SH3 domains, and a C-terminal guanylate kinase domain. Many potential activities for CASK have been suggested, including functions in scaffolding the synapse, organizing ion channels, and regulating neuronal gene transcription [26]. CASK-deficient mice die at first day after birth with neuronal/synaptic defects [18].

GPR34 and GPR82 genes lie, in antisense orientation, in one of the introns of the CASK gene; a localization preserved for 450 million years of vertebrate evolution [27], [28], [29]. Both GPCR are so-called orphan receptors with unknown agonists and physiology. To elucidate their functional relevance we generated gene-deficient mouse lines for both orphan GPCR and performed in-depth phenotypic analyses. An altered CASK function due to confounding effects of the transgenes was almost excluded in GPR34-deficient [19] and GPR82-deficient mice (see *Results*).

GPR34-deficient mice are vital and do not display any obvious differences in morphology, histology, laboratory chemistry or behavior in a standard laboratory environment compared to their WT littermates [19]. Examinations under different immune system-challenging conditions revealed that GPR34 deficiency interferes with proper immune response and results in a reduced resistance to systemic infections.

GPR82-deficient mice showed distinct phenotypes in some neuronal functions (motor activity, hot plate test) and a reduced immune response (DTH test). These tests were performed since GPR82 showed reasonable expression in brain, microglia, spleen and bone marrow-derived dendritic cells (data not shown). Future studies will more specifically address the relevance of GPR82 e.g. in nociception and in immune cell function. However, the most obvious phenotype was the reduced body weight. It is widely accepted that an imbalance between food intake and energy expenditure and, subsequently the deposition of excess fatty acids into fat cells, leads to an increase in body weight and finally to consequences of obesity such as the metabolic syndrome [30], [31]. GPR82 deficiency results in the opposite phenotype with a reduced body and fat mass, a higher glucose tolerance and lower plasma TAG levels. Low food intake, increased physical activity or a higher basal metabolic rate may account for such phenotypes. Increased activity did not account for the phenotype of GPR82-deficient mice because KO mice were less active during the dark period compared to WT mice (Figure 5B). In concert with this observation KO mice had lower total and spontaneous activities, a reduced velocity and moved a shorter distance in open field and light/dark tests compared to their WT littermates (*Table S7*). However, GPR82 deficiency results in a significantly reduced food intake (Figure 5C). Because the decreased food intake lasted the entire 24-h observation period, the reduced motor activity found during the dark period cannot fully account for the reduced feeding activity.

Since GPR82 is expressed in the hypothalamus (*Table S1*), one can speculate that this GPCR is also involved in central appetite regulation. However, different diets had no effect on GPR82 expression and genes related to regulation of food intake showed no significant expression changes in GPR82-deficient mice. Further studies with conditional GPR82-deficient mice may determine its specific role in the hypothalamus. At this point the mechanism, how GPR82 deficiency leads to reduced food intake, remains speculative. However, all peripheral phenotypes, such as reduced fat content (Figure 1), lower serum TAG levels (Figure 2), lower glucose levels in tolerance tests (Figure 3), can simply be interpreted as secondary effects to the reduced energy uptake. This is supported by the fact that reduced fat mass in KO mice is exclusively caused by a lower TAG/water ratio (*Figure S7*) and smaller adipocytes (Figure 1D) but not by differences in the fat composition (*Figure S8*).

But is the reduced body weight exclusively a result of a reduced food intake? GPR82-deficient mice present slightly elevated fT4 serum levels (*Table S5*) and an increased body temperature (*Figure S9*). Similarly, mice with transgenically induced hyperthyroidism (increased fT3 and fT4) displayed reduced body weight (about 10–15%) and increased body temperature (0.4°C) [32], [33]. Therefore, it cannot be completely excluded that the elevated fT4 levels, as a measure for a higher thyroid hormone activity, contribute to the phenotype at least in male KO mice. However, reduced motor activity is rather unusual in hyperthyroidism and thyroid hormone exerts negative feedback on hypothalamic type 4 melanocortin receptor expression resulting in an increase of appetite [34]. It appears that the effect of GPR82 on locomotion involves additional neuronal mechanisms that need to be addressed in future studies. To further distinguish appetite from metabolic mechanisms, food clamp, and cold exposure tests or, more directly, measurements of the energy balance are helpful. No differences in the respiratory and metabolic rates were found between WT and KO mice almost excluding metabolic- and temperature-driven effects on body weight. These data support our hypothesis that reduced food intake is the main cause of lower body weight in GPR82-deficient mice.

In human males, SNP rs660915 and rs4827286 showed significant association with BMI and explained BMI differences of 0.4 and 0.6, respectively, in a meta-analysis of three human cohorts (see *Tables S11, S12*). However, the genotype-BMI association in both Leipzig cohorts was not seen when these cohorts were analyzed separately (*Table S12*). The finding is not unexpected keeping in mind that the phenotypes identified in mice are the result of a complete gene deficiency. Further, the human cohorts are genetically more heterogeneous than the inbred mouse strain and epistatic effects may influence the quantity of BMI, which is per se a very convergent phenotype. Sequencing of the human GPR82-coding region of the major European haplotypes revealed no variants except of a rare silent mutation at Asp^313^Asp. Therefore, phenotypical differences are probably related to sequence differences at the GPR82 locus which may participate in gene regulation.

### Conclusion

We found clear evidence that the CASK locus, which contains two orphan GPCR, GPR34 and GPR82, underwent recent selection in Asian and European populations. Although GPR82 is not required for essential vital function of vertebrates, our findings with gene-deficient mice suggest an involvement of GPR82 in food intake, glucose and lipid homeostasis and, finally, body weight. It appears that GPR82 function is required for optimal energy accumulation making this orphan GPCR a candidate gene for the ‘thrifty gene’ hypothesis. The fundamental basis of the hypothesis is that, in evolutionary history, genes (or gene variants) promoting efficient fat deposition could have been advantageous because they allowed their holders to survive periods of famine. In modern human society, such genes or their variants are disadvantageous because they promote fat deposition in preparation for a never emerging famine, resulting in widespread obesity and diabetes. Therefore, the impact of GPR82 function on energy homeostasis under specific conditions, e.g. diabetes and atherosclerosis, requires further investigation. Identification of the endogenous agonist of GPR82 is of great importance and, based on our findings with GPR82-deficient mice, the development of antagonists may be advantageous to reduce food intake and body fat.

It should finally be stated that our data provide no proof that the signatures of recent selection at the CASK locus are directly related to GPR82 and its function in modulating energy and lipid metabolism. The specific functions of the two other genes, CASK and GPR34, may have also triggered the recent fixation of only one major haplotype, e.g. in the Asian population. The involvement of GPR82 and its relevance in energy homeostasis is one reasonable scenario. Further studies are needed to proof or reject hypotheses about the selective forces that produced the found footprint of selection at this genomic region.

## Materials and Methods

If not stated otherwise all standard substances were purchased from Sigma Aldrich (Taufkirchen, Germany), Merck (Darmstadt, Germany), and C. Roth GmbH (Karlsruhe, Germany). Cell culture material and primers were obtained from Invitrogen (Karlsruhe, Germany). Primer sequences will be provided on request.

### Ethics Statement

All animal experiments were conducted in accord with accepted standards of humane animal care and approved by the respective regional government agency of the State of Saxony, Germany (TVV 43/07). Genetic investigations on humans were conducted in agreement with the patient according to Declaration of Helsinki principles.

### Genotyping of Variants in Human Populations and Selection Analysis

Scanning SNP data for signals of recent selection using data from the International HapMap Project revealed significant signatures for the CASK locus [6] (http://hg-wen.uchicago.edu/selection/haplotter.htm). To further verify these initial findings with other statistical methods SNP data (100 kbp up- and downstream of the GPR34 start ATG) from 154 unrelated individuals were retrieved from the HapMap database (Phase II) [35] (see *Methods S1*). Additional SNP data from 427 (252 female, 175 male) individuals of a local Sorbs cohort [20] were retrieved from 500 K Affymetrix GeneChip analyses. For analysis of the Sorbs cohort only individuals with identity by descent (IBD) sharing <0.15 were included. Approximately 10 kbp of the CASK locus were amplified and sequenced in 50 male individuals (23 Asians: 16 Han Chinese (HCB) from HGDP-CEPH panel, 6 Koreans and 1 Vietnamese; 27 Sub-Saharan Africans: Yoruba (YRI), Mandenka, Mbuti Pygmies from HGDP-CEPH panel).

All SNP and sequence data sets were analyzed for signatures of positive selection applying the D-tests of Tajima [36] and Fu and Li [37] using DnaSP v5 software [38].

### Genotype-Phenotype Association Studies

Seven tagging SNP were retrieved from a 55-kb genomic region (X:41,430,000–41,485,000, NCBI Build 36), including GPR34 and GPR82, in the HapMap European population (CEU) data set using the application “Tagger” implemented in “Haploview 4.1” [39] (see *Figure S10*). Genotype data for the tagging SNP rs913602 in the Sorbs cohort were retrieved from the data set generated by genotyping 937 individuals using 500 K Affymetrix GeneChip Arrays [20]. The remaining 6 tagging SNP were successfully genotyped in 1,014 individuals of the Sorbs cohort [20] using the TaqMan assay (Applied Biosystems, Inc., oligonucleotide sequences available upon request). TaqMan genotyping was performed according to the manufacturer's protocol on an ABI PRISM 7500 sequence detector (Applied Biosystems Inc.). Genotype success rate was 97%. To assess genotyping reproducibility a random 5% selection of the samples was re-genotyped for each SNP; all genotypes matched initial designated genotypes. Two additional local cohorts, Leipzig [24] and Leipzig Heart Study [25], were genotyped at two tagging SNP sites (rs6609159, rs4827286) using the same TaqMan assay. Associations between genotypes (independent variables) and different clinical and laboratory chemical parameters (dependent variables) were assessed by linear regression analysis with adjustment for age and - except for BMI, body fat and lean body mass content - BMI. All analyses were performed stratified by gender. Because males are hemizygous only female individuals homozygous for a SNP variant were included in the analysis. Non-normally distributed parameters were ln-transformed. Analyses were performed using SPSS, version 15.0 for Windows (Chicago, IL, USA). Further, sequence database source (NCBI, HapMap) and genomic DNA collections of human populations with African, Asian, and European ancestry were screened for GPR82 variants. Thus, PCR were performed using a specific primer pair flanking the human GPR82 and the coding sequence of GPR82 was sequenced.

### Tissue Expression Studies

The genomic structure of the mouse GPR82 was determined by analyzing mouse GPR82 transcripts. Thus, RNA was isolated from 30 mg mouse epididymis using the SV Total RNA isolation system (Promega, Mannheim, Germany). For amplification of the 5′ cDNA ends the GeneRacer Kit (Invitrogen, Carlsbad, CA, USA) was used according to the manufacturer's protocol. PCR products were cloned into the pCR4-TOPO vector and 27 clones were sequenced. Several 5′ UTR exons were identified. This information was used for primer design to prevent genomic GPR82 amplificates in expression analyses.

For qualitative expression analysis several mouse tissues were dissected and RNA was isolated from 60 mg tissue using TRIZOL (Invitrogen) according to the manufacturer's protocol. Complementary DNA was synthesized from 1 µg of total RNA with reverse transcriptase (Superscript II kit, Invitrogen) and an oligo-dT primer.

Quantitative PCR (qPCR) was performed by the Platinum SYBRgreen qPCR Super-Mix-UDG (Invitrogen) according to the manufacturer's protocol. To exclude genomic amplification primers were complementary to GPR82 exon sequences flanking introns. The point at which the amplification plot crossed the threshold was defined as C_t_ value which represented the cycle number at this point. Products were quantified to the transcript levels of the housekeeping gene β2 microglobulin by normalizing C_t_ values to the control and given as ΔC_t_ (ΔC_t_ = C_t(GPR82)_−C_t(β2-microglobulin)_).

### Generation of a GPR82-deficient Mouse Model and Phenotypical Characterization

GPR82-deficient (KO) mice were kindly provided by Ingenium AG, Martinsried, Germany. Mice were generated by homologous recombination (for details see *Methods S1*, *Figure S2*). The mice background was C3HeB/FeJ and animals of generations >12 were used for the study. The different genotypes and genders were generated by intercrossing the respective heterozygous and homozygous animals (because of X-chromosomal location). Routine genotyping of mice was performed by multiplex PCR (see *Methods S1*).

The genotype distribution of all litters from 17 and 32 matings of heterozygous females with WT and KO male animals was determined. The body weight development was recorded for a period of 3 months. Eight WT and 11 KO male mice were tested in an adapted SHIRPA protocol [40], [41] (for experimental details see http://empress.har.mrc.ac.uk/browser). More animals were tested in further phenotyping screens, including morphological and histological studies, blood analysis, hot plate test, magnetic resonance measurements, motor activity, feeding, respiratory and metabolic rates measurements and delayed-type hypersensitivity test (DTH) (for experimental details see *Methods S1*). At the age of 12 weeks an oral glucose tolerance test (oGTT) was performed (see *Methods S1*). Fasting plasma insulin was measured by ELISA (Crystal Chem, Downers Grove, IL, USA). NMR and MALDI-TOF investigations on brown, perigonadal and subcutaneous fat tissue were performed as described [42], [43] (see *Methods S1*). If not stated otherwise statistical analyses were performed using the Mann-Whitney test. Standard diet was: Ssniff M-Z, 5% sugar, 4.5% raw fat, 34% starch, 22% raw protein and Western diet was: Ssniff EF R/M TD88137, 32.8% sugar, 21.2% raw fat, 14.5% starch, 17.1% raw protein.

### Data deposition

New sequences reported in this paper have been deposited in the GenBank database (accession no. FJ789817–FJ789823).

## Supporting Information

Figure S1 **Genomic organization of GPR82 in different species and its transcription start.** To determine the gene structure of GPR82 in mouse (**A**) and human (**B**) specific mRNA and EST sequences were analyzed and compared with genomic sequences. In contrast to humans where two non-coding exons could be found 5′ of the coding sequence, the mouse GPR82 comprises three 5′ non-coding exons (exon 1–3). The figure shows the borders of exons and introns whereas exon sequences are capitalized and the intron sequences are lowercased referred to the first putative translation start codon. Non-coding regions are highlighted in grey, the coding GPR82 sequence is shown in black. Transcript sequencing of a mouse epididymis cDNA library showed that there are four splice variants (1–4). Variant 1 and 3 consist of a shorter exon 1. Additionally, the short intron between exon 3 and 4 is not spliced in variants 3 and 4. (**C**) An RNA ligase-mediated rapid amplification of 5′ cDNA ends (RLM-RACE) approach was used to search for the transcription start of mouse GPR82 as described in *Methods*. By sequencing of 27 cloned sequences the region of the transcription start could be localized containing two TATA-boxes at position −3907 and −3882 as well as a CCAAT box at position −3876 5′ of the 1^st^ non-coding exon. Black dots mark the transcription start of the sequenced clones. The underlined part shows the 1^st^ non-coding exon given by the database.
(TIF)

Figure S2 **Generation and genotyping of GPR82-deficient mice.** (**A**) The GPR82 KO mouse was made on a C3HeB/FeJ background by a conventional KO strategy. A part of the mouse exon 4 containing the GPR82 coding sequence (cds) was replaced by a cassette containing an EMCV-IRES site, the *E. coli* lacZ gene encoding β-galactosidase and the neomycin resistance gene. The non-coding sequences are highlighted in grey whereas the coding sequence is shown in black. The primers for genotyping of littermates are displayed as **a** (5′-TTCTCTTGTCAGCCATCTGC-3′), **b** (5′-AACATCCTCACTTGTCTTGCA-3′) and **c** (5′-AGAAGGCGATAGAAGGCGAT-3′). **B**) DNA samples from WT, KO and heterozygous mice were amplified with specific primers to yield products of 232 bp, 272 bp or both, respectively. PCR products were separated on 2.5% agarose gels, stained with ethidium bromide, and visualized by ultraviolet (UV) illumination. +/d heterozygous, NTC no template control. **C**) Because the GPR82 gene lies within an intron of the CASK gene and the targeting construct may affect CASK expression, CASK mRNA levels were quantified in different tissues of WT and KO. Oligonucleotide primers (ctacatgagacagatactggaa, ccaagtttaacaggtgccgagt) were designed to flank an intron of the CASK gene and to produce a 123-bp fragment (figure). The mRNA levels of CASK transcripts were quantified by SYBR-Green® real time PCR assays relative to the house keeping gene β2 microglobulin. CASK expression is presented as ΔC_t_ value (table). Data are mean ± SD of 5 animals performed in duplicate. No significant differences in CASK expression levels were found between WT and KO.
(TIF)

Figure S3 **Offspring genotype distribution in GPR82 mouse litters.** Due to the X-chromosomal localization of GPR82 litter distribution was assessed using two different breeding approaches. Heterozygous females were bred with WT males (*n* = 17 litters, open bars) to obtain WT and heterozygous females. To produce homozygous KO female offspring heterozygous females were bred with hemizygous males (*n* = 32 litters, filled bars). There was no significant difference in the number of offspring/litter between the two different matings: 7.41±1.97 (*n* = 126 pups, 17 litters) vs. 6.88±2.17 (*n* = 220 pups, 32 litters). Genotype distribution in litters from matings of heterozygous females with WT males showed a shift towards the recombinant allele. Results are given as mean ± SEM; +/+ WT females, −/+ heterozygous females, −/− KO females, +/Y WT males, −/Y KO males. *** *P*<0.001.
(TIF)

Figure S4 **Hot plate test at 52°C.** A Hot plate test was performed using a Hot Plate 602001 (TSE Technical & Scientific Equipment GmbH, Bad Homburg, Germany). WT (open bars, male *n* = 29, female *n* = 19) and KO (filled bars, male *n* = 21, female *n* = 17) mice were placed into the apparatus and the time lack until shaking or licking one of the hind paws or jumping was recorded. Reaction times are given as mean ± SEM. +/+ WT females, −/− KO females, +/Y WT males, −/Y KO males. ***P<0.001.
(TIF)

Figure S5 **Body weight development of WT and GPR82-deficient mice.** Development of body weight from 4- to 14-week-old female (**A**) and male mice (**B**). The body weights show a significant difference of 10 to 15% between WT (open dots) and KO (filled dots). Challenging the animals with Western diet lead to higher body weights in female (**C**) and male mice (**D**) but only the weight differences between WT and KO male mice stayed highly significant. Body weights are given as mean ± SD. Statistical analysis was performed using the Mann-Whitney test. *P<0.05.
(TIF)

Figure S6 **Intraperitoneal glucose tolerance test (ipGTT).** IpGTT was performed after starving over night by i.p. application of 2 mg glucose/g body weight. Both genotypes (WT data are given as open dots, KO data as filled dots) were fed with Western diet for 12 weeks. For ipGTT the blood glucose concentrations of 13 male KO and 14 WT animals (**A**) and 14 female KO and 17 WT animals (**B**) were compared. Results are given as mean ± SEM. For comparison with oGTT see Fig. 3 C/D of the main text. *P<0.05, **P<0.01, ***P<0.001.
(TIF)

Figure S7 **NMR analysis of the composition of mouse fat tissues under standard and Western diet in WT and KO mice.** ^1^H HRMAS spectra of subcutaneous fat from WT (**A**) and KO mice (**B**) on a Western diet. The NMR spectra show the typical aliphatic signals for fatty acids as well as for glycerol in triacylglycerides. Assignment is given according to [42]. The NMR spectra of the fat tissue from WT mice show significantly higher fat-to-water ratios than KO mice. (**C**) ^1^H HRMAS NMR spectra of brown, subcutaneous and visceral (perigonadal) adipose tissue were recorded for 5 WT (open bars) and KO (filled bars) animals for each diet at a MAS frequency of 9 kHz. Spectral integrals of the CH_2_ (at 1.30 ppm) and the CH_3_ (0.88 ppm) signals were normalized by the integral of the water peak (at 4.65 ppm). Therefore, the water peak shows a normalized integral of unity and the CH_2_/CH_3_ ratio is always approximately constant. Results are given as mean ± SD. *P<0.05.
(TIF)

Figure S8 **Triacylglyceride composition in different fat tissues.** A piece of brown, visceral (perigonadal) and subcutaneous adipose tissue was extracted with chloroform/methanol and subsequently analyzed by positive ion MALDI-TOF mass spectrometry. DHB was used as matrix because this matrix provides the best sensitivity for the detection of apolar compounds [44]. The triacylglyceride composition of 5 male and female animals of each genotype was analyzed. Additionally, mice kept on standard and Western diet were compared. Since no internal standards were used, all intensities are related to the peak with the highest intensity.
(TIF)

Figure S9 **Body temperature.** Transponders for temperature measurement were inserted between shoulder blades of 10 WT and 13 KO anesthetized 12-week-old male animals one week prior to temperature measurement. Temperature was measured at 8 a.m., 11 a.m. and 2 p.m. in duplicates by a DAS 5001 (BioMedic Data Systems, Inc.). The measurements were repeated at the following two days and the mean for the individual animal at each time point was calculated. Temperatures at the three time points of the WT and KO group are given as mean ± SEM. Additionally, all temperature values of the three time points (over all, o.a.) were pooled for WT and for KO animals. *P* values are given.
(TIF)

Figure S10 **Identification and localization of tagging SNP nearby GPR34 and GPR82.** We searched the HapMap data set of the European population (CEU) for tagging SNP in a 55-kb genomic region (X: 41,430,000–41,485,000, NCBI Build 36) spanning the GPR34 and GPR82 locus using application “Tagger” in “Haploview 4.1” [39]. Seven tagging SNP cover common genetic variations (minor allele frequency >0.05, r^2^>0.8) in this genomic area. Numbers in grey fields are r^2^ values (multiplied by 100).
(TIF)

Table S1 **Expression of the mouse GPR82 determined by qPCR.** WT mice were sacrificed and total RNA was isolated from all major tissues. The mRNA levels of GPR82 transcripts were quantified by SYBR-Green® real time PCR assays relative to the house keeping gene β2 microglobulin. GPR82 expression is presented as x-fold over GPR82 expression in liver as the organ with the lowest expression level (ΔC_t_: 19.44±0.95) using the 2^−ΔΔCt^ method [7]. Data are mean ± SD of 5 independent experiments performed in triplicate. GPR82 was not detected in different embryonic stages. Data significantly different from the expression level in liver are marked with *P<0.05; **P<0.01; ***P<0.001.
(DOC)

Table S2 **Organ weight.** Organs from 3-month-old male and female mice were removed and weighed. (**A**) Weight per organ is given in gram. Results are mean ± SD. (**B**) Weight per organ is given in % of whole body weight. Results are mean ± SD. *P<0.05; **P<0.01; ***P<0.001.
(DOC)

Table S3 **Hemogram of GPR82-deficient mice.** Whole blood samples from three-month-old mice were analyzed by an automatic hemocytometer (ScilVet ABC; scil animal care company GmbH, Viernheim, Germany). Results are given as mean ± SEM. *P<0.05, **P<0.01, ***P<0.001, significant different parameters (MCV, MCHC, RDW) are still in the normal range.
(DOC)

Table S4 **AA/AC screening of whole blood samples.** In the table listed amino acids and acyl carnitines were measured using an ESI-MS/MS from dried blood samples. Results are given as mean ± SEM. *P<0.05.
(DOC)

Table S5 **Phenotypical and laboratory screen for differences between WT and KO mice under standard diet.** 15-week-old male and female WT and KO mice were subjected to different analyses, including QMR to determine the body composition, an oGTT to test for the answer to a glucose application, determination of insulin concentrations in serum, serum analysis for cholesterol and TAG levels and clinical chemistry parameters. Results are given as mean ± SD. *P<0.05; **P<0.01; ***P<0.001.
(DOC)

Table S6 **Phenotypical and laboratory screen for differences between WT and KO mice under Western-type diet.** 15-week-old male and female WT and KO mice were kept under Western diet for 12 weeks and subjected to different analyses as mice under normal diet (see *suppl. Table S3*). Results are given as mean ± SD. *P<0.05, **P<0.01, ***P<0.001.
(DOC)

Table S7 **Results of SHIRPA Protocol.** Following the modified SHIRPA protocol [8], [9] WT and KO male mice were tested. Results are means of scores from 3 independent observers ± SD (http://phenome.jax.org/pub-cgi/phenome/mpdcgi?rtn=projects/docstatic&doc=Lake2/Lake2_Protocol). Open field and light/dark test: 3-month-old male mice were tested in the setup. Recording and analysis was performed automatically. The table shows main parameters as mean ± SD. *P<0.05; **P<0.01.
(DOC)

Table S8 **DTH test.** WT and KO male animals were intradermally immunized by methylated BSA emulsified in CFA. At day 8 p.i. mBSA was injected intradermally into one hind paw and 0.9% saline into the other hind paw as a control. Paw swelling was measured at time points as indicated. For details see *suppl. Methods*. *P<0.05; **P<0.01; ***P<0.001
(DOC)

Table S9 **Expression of selected genes involved in regulation of energy balance and food intake.** Gene expression ratios are given as ΔC_t_ values (mean ± SEM) with the quantitative (q) PCR sample size in parentheses. The mean C_t_ values of β2 microglobulin in stomach (WT: 20.3±0.2 *vs.* KO: 19.5±0.1), hypothalamus (WT: 20.8±0.1 *vs.* KO: 21.5±0.1), and liver (WT: 18.9±0.7 *vs.* KO: 20.0±0.2) were not significantly different between WT and KO. n.d. not determined, *P<0.05.
(DOC)

Table S10 **Determination of the energy balance in metabolic cages.** 20-week-old male mice were adapted to metabolic cages (Phenomaster, TSE Systems) and monitored over 24 h. Values are given as mean ± SD. *P<0.05; **P<0.01; ***P<0.001.
(DOC)

Table S11 **Phenotype-genotype correlation in the Sorbs cohort – Genotype frequency distribution.** (**A**) ∼900 individuals (*for details see Experimental Procedures*) of the self-contained population of Sorbs were genotyped at 7 tagging SNP sites covering the genes for GPR34 and GPR82. Only homozygous female individuals were included in the association analysis (because males are hemizygous). Number of individuals homo-/hemizygous for the allele are given. (**B**) All effect directions (beta) in the association analysis were standardized to the minor allele and are shown with corresponding p-values. Only non-diabetic individuals and, for female individuals, only homozygotes (because males are hemizygous) were included. Associations with serum lipid parameters were assessed only in homo-/hemizygous individuals without lipid-lowering medication. Data are displayed for both genders. *P<0.05; **P<0.01.
(DOC)

Table S12 **BMI-genotype correlation at tagging SNP rs6609159 and rs4827286.** (A, B) ∼530 individuals of a cohort from Leipzig [10] were genotyped at two tagging SNP sites (rs6609159, rs4827286) that showed strongest association with BMI in the Sorbs cohort (see above). (C, D) ∼1900 individuals of the Leipzig Heart Study [11] were genotyped at two tagging SNP sites (rs6609159, rs4827286). (E, F) Meta analysis of the two additional cohorts together with the Sorbs cohort (∼3330 individuals) revealed significant correlations between genotypes and BMI. For female individuals only homozygous individuals were included in the association analysis (because males are hemizygous). Numbers of individuals homo-/hemizygous for the allele are given in A, C, E). All effect directions (beta) in the association analysis were standardized to the minor allele and are shown with corresponding p-values (B, D, F). *P<0.05; **P<0.01.
(DOC)

Methods S1 **Supplementary Methods.**
(DOC)
