# Supplementary figures and images for: Reduced Food Intake and Body Weight in Mice Deficient for the G Protein-Coupled Receptor GPR82
Source: PLoS One. 2011 Dec 28;6(12):e29400. doi: 10.1371/journal.pone.0029400 (PMC3247265; doi:10.1371/journal.pone.0029400)

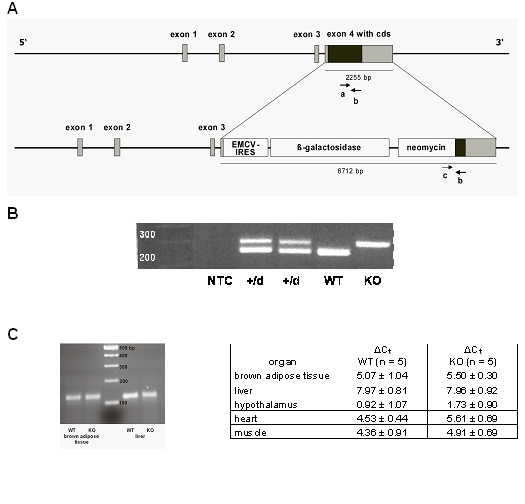

Supplement: Figure S2 — Generation and genotyping of GPR82-deficient mice. (A) The GPR82 KO mouse was made on a C3HeB/FeJ background by a conventional KO strategy. A part of the mouse exon 4 containing the GPR82 coding sequence (cds) was replaced by a cassette containing an EMCV-IRES site, the E. coli lacZ gene encoding β-galactosidase and the neomycin resistance gene. The non-coding sequences are highlighted in grey whereas the coding sequence is shown in black. The primers for genotyping of littermates are displayed as a (5′-TTCTCTTGTCAGCCATCTGC-3′), b (5′-AACATCCTCACTTGTCTTGCA-3′) and c (5′-AGAAGGCGATAGAAGGCGAT-3′). B) DNA samples from WT, KO and heterozygous mice were amplified with specific primers to yield products of 232 bp, 272 bp or both, respectively. PCR products were separated on 2.5% agarose gels, stained with ethidium bromide, and visualized by ultraviolet (UV) illumination. +/d heterozygous, NTC no template control. C) Because the GPR82 gene lies within an intron of the CASK gene and the targeting construct may affect CASK expression, CASK mRNA levels were quantified in different tissues of WT and KO. Oligonucleotide primers (ctacatgagacagatactggaa, ccaagtttaacaggtgccgagt) were designed to flank an intron of the CASK gene and to produce a 123-bp fragment (figure). The mRNA levels of CASK transcripts were quantified by SYBR-Green® real time PCR assays relative to the house keeping gene β2 microglobulin. CASK expression is presented as ΔCt value (table). Data are mean ± SD of 5 animals performed in duplicate. No significant differences in CASK expression levels were found between WT and KO. (TIF) [file pone.0029400.s002.tif]
