# Supplementary material for: Reduced Food Intake and Body Weight in Mice Deficient for the G Protein-Coupled Receptor GPR82
Source: PLoS One. 2011 Dec 28;6(12):e29400. doi: 10.1371/journal.pone.0029400 (PMC3247265; doi:10.1371/journal.pone.0029400)
Supplement: Table S1 — Expression of the mouse GPR82 determined by qPCR. WT mice were sacrificed and total RNA was isolated from all major tissues. The mRNA levels of GPR82 transcripts were quantified by SYBR-Green® real time PCR assays relative to the house keeping gene β2 microglobulin. GPR82 expression is presented as x-fold over GPR82 expression in liver as the organ with the lowest expression level (ΔCt: 19.44±0.95) using the 2−ΔΔCt method [7]. Data are mean ± SD of 5 independent experiments performed in triplicate. GPR82 was not detected in different embryonic stages. Data significantly different from the expression level in liver are marked with *P<0.05; **P<0.01; ***P<0.001. (DOC) [file pone.0029400.s011.doc]

| ***organ*** | ***mean ± SD (n = 5)*** | ***∆∆C_t_*** | ***2^(-∆∆Ct)^*** |
| --- | --- | --- | --- |
| liver | 19.44 ± 0.95 | 0.00 | 1.00 |
| perigonadal fat | 16.69 ± 1.77 | -2.75 | 6.72 |
| brown fat | 16.32 ± 0.45*** | -3.12 | 8.70 |
| submandibular gland | 16.06 ± 1.70** | -3.38 | 10.44 |
| subcutaneous fat | 15.35 ± 1.35* | -3.72 | 13.14 |
| cerebellum | 15.02 ± 0.66*** | -4.42 | 21.34 |
| heart | 14.95 ± 0.96*** | -4.49 | 22.45 |
| kidney | 14.88 ± 0.62*** | -4.56 | 23.59 |
| ileum | 13.79 ± 1.43*** | -5.65 | 50.07 |
| muscle | 13.30 ± 2.16** | -6.14 | 70.32 |
| duodenum | 13.30 ± 1.39*** | -6.14 | 70.33 |
| jejunum | 13.18 ± 0.73*** | -6.26 | 76.43 |
| lung | 12.68 ± 0.49*** | -6.76 | 108.53 |
| colon | 12.59 ± 1.36*** | -6.88 | 117.46 |
| spleen | 12.36 ± 0.77*** | -7.08 | 135.42 |
| cerebrum | 11.93 ± 1.24*** | -7.51 | 182.36 |
| hypothalamus | 11.70 ± 1.27*** | -7.74 | 214.23 |
| testis | 9.02 ± 1.04*** | -10.42 | 1366.24 |
| epididymis | 6.47 ± 0.98*** | -12.97 | 8027.12 |
