# Supplementary material for: Reduced Food Intake and Body Weight in Mice Deficient for the G Protein-Coupled Receptor GPR82
Source: PLoS One. 2011 Dec 28;6(12):e29400. doi: 10.1371/journal.pone.0029400 (PMC3247265; doi:10.1371/journal.pone.0029400)
Supplement: Table S2 — Organ weight. Organs from 3-month-old male and female mice were removed and weighed. (A) Weight per organ is given in gram. Results are mean ± SD. (B) Weight per organ is given in % of whole body weight. Results are mean ± SD. *P<0.05; **P<0.01; ***P<0.001. (DOC) [file pone.0029400.s012.doc]

| **A** | ***male*** | | | ***female*** | |
| --- | --- | --- | --- | --- | --- |
|  | ***WT (n = 31)*** | ***KO (n = 23)*** | ***WT (n = 20)*** | | ***KO (n = 31)*** |
| body weight | 29.91 ± 3.29 | 25.97 ± 2.55 *** | 25.64 ± 3.09 | | 23.44 ± 2.69 * |
| **organ** |  |  |  | |  |
| liver | 1.43 ± 0.16 | 1.34 ± 0.18 | 1.26 ± 0.21 | | 1.20 ± 0.17 |
| brain | 0.45 ± 0.03 | 0.41 ± 0.03 | 0.46 ± 0.04 | | 0.43 ± 0.02 ** |
| left kidney | 0.30 ± 0.03 | 0.27 ± 0.03 | 0.19 ± 0.02 | | 0.18 ± 0.02 |
| right kidney | 0.28 ± 0.03 | 0.25 ± 0.03 | 0.17 ± 0.02 | | 0.17 ± 0.02 |
| seminal vesicle | 0.18 ± 0.03 | 0.14 ± 0.05 | --- | | --- |
| lung | 0.16 ± 0.03 | 0.17 ± 0.03 | 0.16 ± 0.02 | | 0.15 ± 0.02 |
| testis right | 0.13 ± 0.02 | 0.12 ± 0.02 | --- | | --- |
| testis left | 0.11 ± 0.01 | 0.11 ± 0.01 | --- | | --- |
| heart | 0.12 ± 0.01 | 0.12 ± 0.01 | 0.11 ± 0.01 | | 0.10 ± 0.01 |
| spleen | 0.09 ± 0.01 | 0.09 ± 0.01 | 0.12 ± 0.02 | | 0.11 ± 0.03 |
| brown fat | 0.08 ± 0.02 | 0.08 ± 0.01 | 0.07 ± 0.01 | | 0.07 ± 0.02 |
| urinary bladder | 0.03 ± 0.00 | 0.03 ± 0.01 | 0.02 ± 0.00 | | 0.02 ± 0.01 |
| uterus | --- | --- | 0.14 ± 0.03 | | 0.14 ± 0.03 |

| **B** | ***male*** | | ***female*** | |
| --- | --- | --- | --- | --- |
|  | ***WT (n = 31)*** | ***KO (n = 23)*** | ***WT (n = 20)*** | ***KO (n = 31)*** |
| body weight | 29.91 ± 3.29 | 25.97 ± 2.55 *** | 25.64 ± 3.09 | 23.44 ± 2.69 * |
| **organ** |  |  |  |  |
| liver | 4.79 ± 0.40 | 5.14 ± 0.50 ** | 4.90 ± 0.38 | 5.16 ± 0.70 |
| brain | 1.51 ± 0.15 | 1.58 ± 0.14 | 1.81 ± 0.21 | 1.85 ± 0.18 |
| kidney left | 1.01 ± 0.07 | 1.04 ± 0.08 | 0.73 ± 0.05 | 0.76 ± 0.08 |
| kidney right | 0.94 ± 0.08 | 0.99 ± 0.07 * | 0.67 ± 0.09 | 0.71 ± 0.08 |
| seminal vesicle | 0.63 ± 0.11 | 0.56 ± 0.18 | --- | --- |
| lung | 0.54 ± 0.07 | 0.67 ± 0.14 *** | 0.63 ± 0.08 | 0.65 ± 0.10 |
| testis right | 0.42 ± 0.06 | 0.42 ± 0.05 | --- | --- |
| testis left | 0.41 ± 0.06 | 0.41 ± 0.06 | --- | --- |
| heart | 0.40 ± 0.04 | 0.44 ± 0.05 *** | 0.42 ± 0.04 | 0.44 ± 0.06 |
| spleen | 0.29 ± 0.04 | 0.33 ± 0.04 *** | 0.45 ± 0.06 | 0.48 ± 0.14 |
| brown fat | 0.26 ± 0.07 | 0.29 ± 0.07 | 0.27 ± 0.05 | 0.30 ± 0.09 |
| urinary bladder | 0.09 ± 0.01 | 0.11 ± 0.02 * | 0.08 ± 0.02 | 0.10 ± 0.05 |
| uterus | --- | --- | 0.55 ± 0.12 | 0.62 ± 0.14 |
