# Supplementary material for: Reduced Food Intake and Body Weight in Mice Deficient for the G Protein-Coupled Receptor GPR82
Source: PLoS One. 2011 Dec 28;6(12):e29400. doi: 10.1371/journal.pone.0029400 (PMC3247265; doi:10.1371/journal.pone.0029400)
Supplement: Table S3 — Hemogram of GPR82-deficient mice. Whole blood samples from three-month-old mice were analyzed by an automatic hemocytometer (ScilVet ABC; scil animal care company GmbH, Viernheim, Germany). Results are given as mean ± SEM. *P<0.05, **P<0.01, ***P<0.001, significant different parameters (MCV, MCHC, RDW) are still in the normal range. (DOC) [file pone.0029400.s013.doc]

|  | ***female*** | |
| --- | --- | --- |
| ***blood parameters*** | ***WT (n = 22)*** | ***KO (n = 10)*** |
| WBC (10³/mm³) | 8.44 ± 0.48 | 8.93 ± 0.58 |
| RBC (10^6^/mm³) | 7.25 ± 0.21 | 7.26 ± 0.54 |
| HGB (g/dl) | 14.98 ± 0.41 | 14.04 ± 0.37 |
| HCT (%) | 41.90 ± 0.62 | 42.67 ± 1.04 |
| PLT (10³/mm³) | 984.4 ± 50.3 | 990.4 ± 72.3 |
| MCV (µm³) | 57.77 ± 0.23 | 58.80 ± 0.23 ** |
| MCH (pg) | 20.46 ± 0.65 | 19.32 ± 0.10 |
| MCHC (g/dl) | 35.44 ± 1.22 | 32.87 ± 0.14 * |
| RDW (%) | 12.63 ± 0.37 | 14.23 ± 0.15 *** |
| MPV (µm³) | 5.66 ± 0.09 | 5.87 ± 0.13 |
| LYM (%) | 67.32 ± 0.97 | 68.29 ± 2.09 |
| LYM (10³/mm³) | 5.49 ± 0.27 | 5.99 ± 0.31 |
| MONO (%) | 5.05 ± 0.24 | 4.97 ± 0.46 |
| MONO (10³/mm³) | 0.37 ± 0.03 | 0.39 ± 0.04 |
| GRAN (%) | 27.69 ± 1.08 | 27.64 ± 2.20 |
| GRAN (10³/mm³) | 2.43 ± 0.18 | 2.55 ± 0.33 |
