# Supplementary material for: Reduced Food Intake and Body Weight in Mice Deficient for the G Protein-Coupled Receptor GPR82
Source: PLoS One. 2011 Dec 28;6(12):e29400. doi: 10.1371/journal.pone.0029400 (PMC3247265; doi:10.1371/journal.pone.0029400)
Supplement: Table S4 — AA/AC screening of whole blood samples. In the table listed amino acids and acyl carnitines were measured using an ESI-MS/MS from dried blood samples. Results are given as mean ± SEM. *P<0.05. (DOC) [file pone.0029400.s014.doc]

|  | ***male*** | |
| --- | --- | --- |
| ***amino acids (µmol/l)*** | ***WT (n = 16)*** | ***KO (n = 31)*** |
| Ala | 269.02 ± 16.22 | 284.80 ± 13.52 |
| Arg | 88.34 ± 8.95 | 99.32 ± 7.17 |
| Asp | 79.17 ± 4.86 | 87.21 ± 4.87 |
| Cit | 40.49 ± 4.29 | 45.38 ± 3.41 |
| Glu | 188.49 ± 7.53 | 208.02 ± 9.69 |
| Gly | 230.72 ± 12.81 | 243.37 ± 10.17 |
| His | 143.21 ± 12.35 | 155.54 ± 14.36 |
| Leu/Ile | 165.17 ± 9.09 | 170.22 ± 6.96 |
| Met | 34.01 ± 2.06 | 40.68 ± 2.41* |
| Orn | 62.54 ± 7.15 | 64.48 ± 7.96 |
| Phe | 65.68 ± 3.78 | 66.07 ± 2.95 |
| Pro | 229.00 ± 19.58 | 207.12 ± 11.41 |
| Ser | 70.47 ± 10.04 | 68.68 ± 6.52 |
| Thr | 82.50 ± 6.96 | 81.41 ± 4.26 |
| Tyr | 66.81 ± 7.21 | 70.13 ± 4.69 |
| Val | 161.40 ± 4.99 | 156.82 ± 6.41 |
| ***acyl carnitines (µmol/l)*** | | |
| C2 | 32.26 ± 1.79 | 36.33 ± 1.68 |
| C4 | 0.41 ± 0.04 | 0.39 ± 0.02 |
| C8 | 0.16 ± 0.02 | 0.15 ± 0.01 |
| C12 | 0.15 ± 0.02 | 0.13 ± 0.01 |
| C14 | 0.23 ± 0.02 | 0.25 ± 0.02 |
| C16 | 1.46 ± 0.06 | 1.36 ± 0.04 |
| C16:1 | 0.12 ± 0.02 | 0.10 ± 0.01 |
| C18 | 0.40 ± 0.02 | 0.39 ± 0.02 |
| C18:1 | 0.53 ± 0.05 | 0.47 ± 0.02 |
