# Supplementary material for: Reduced Food Intake and Body Weight in Mice Deficient for the G Protein-Coupled Receptor GPR82
Source: PLoS One. 2011 Dec 28;6(12):e29400. doi: 10.1371/journal.pone.0029400 (PMC3247265; doi:10.1371/journal.pone.0029400)
Supplement: Table S5 — Phenotypical and laboratory screen for differences between WT and KO mice under standard diet. 15-week-old male and female WT and KO mice were subjected to different analyses, including QMR to determine the body composition, an oGTT to test for the answer to a glucose application, determination of insulin concentrations in serum, serum analysis for cholesterol and TAG levels and clinical chemistry parameters. Results are given as mean ± SD. *P<0.05; **P<0.01; ***P<0.001. (DOC) [file pone.0029400.s015.doc]

|  | ***male*** | | ***female*** | |
| --- | --- | --- | --- | --- |
| ***Parameter*** | ***WT*** | ***KO*** | ***WT*** | ***KO*** |
| ***body composition*** | ***(n = 26)*** | ***(n = 30)*** | ***(n = 10)*** | ***(n = 7)*** |
| body weight | 32.96 ± 2.77 | 29.46 ± 2.00 *** | 31.46 ± 2.75 | 25.63 ± 4.37 *** |
| fat (g) | 3.18 ± 1.88 | 1.35 ± 1.03 *** | 5.11 ± 1.58 | 1.99 ± 1.28 *** |
| fat (% body weight) | 9.29 ± 4.92 | 4.54 ± 3.35 *** | 16.10 ± 4.32 | 7.35 ± 3.60 *** |
| lean (g) | 25.83 ± 1.22 | 24.26 ± 1.88 *** | 22.85 ± 1.59 | 20.24 ± 3.02 ** |
| lean (% body weight) | 78.67 ± 4.35 | 82.4 ± 3.71 *** | 72.84 ± 4.50 | 79.24 ± 2.60 *** |
| water (g) | 23.33 ± 1.09 | 21.96 ± 1.70 *** | 20.61 ± 1.45 | 18.32 ± 2.70 ** |
| water (% body weight) | 64.58 ± 3.44 | 71.06 ± 3.90 *** | 65.72 ± 4.13 | 71.73 ± 2.36 *** |
| ***oGTT*** | ***(n = 25)*** | ***(n = 22)*** | ***(n = 17)*** | ***(n = 10)*** |
| fasting blood glucose | 6.89 ± 0.63 | 6.41 ± 0.68 * | 6.66 ± 0.65 | 6.17 ± 0.56 |
| *blood glucose after* | | | | |
| 20 min | 10.78 ± 2.12 | 9.45 ± 1.91 * | 8.31 ± 1.79 | 8.39 ± 1.12 |
| 40 min | 7.44 ± 1.03 | 6.93 ± 1.12 | 7.38 ± 0.84 | 7.28 ± 0.99 |
| 60 min | 7.29 ± 0.92 | 6.90 ± 0.87 | 7.35 ± 0.59 | 7.27 ± 0.89 |
| 120 min | 6.69 ± 0.88 | 5.83 ± 0.96 ** | 6.05 ± 0.61 | 6.22 ± 0.97 |
| AUC | 926 ± 99* | 843 ± 93 ** | 856 ± 77 | 853 ± 78 |
| ***insulin concentration*** | ***(n = 15)*** | ***(n = 11)*** | ***(n = 12)*** | ***(n = 14)*** |
| insulin (ng/ml) | 0.48 ± 0.10 | 0.41 ± 0.11 | 0.50 ± 0.11 | 0.42 ± 0.09 * |
| ***serum*** | ***(n = 28)*** | ***(n = 40)*** | ***(n = 11)*** | ***(n = 5)*** |
| HDL cholesterol (mmol/l) | 2.51 ± 0.06 | 2.49 ± 0.07 | 1.83 ± 0.10 | 2.15 ± 0.18 |
| HDL triacylglycerides (mmol/l) | 0.86 ± 0.03 | 0.67 ± 0.03 | 0.84± 0.03 | 0.85 ± 0.04 |
| LDL cholesterol (mmol/l) | 1.14 ± 0.02 | 1.10 ± 0.11 | 0.99 ± 0.14 | 1.11 ± 0.15 |
| LDL triacylglycerides (mmol/l) | 1.64 ± 0.08 | 1.28 ± 0.03 *** | 1.88 ± 0.09 | 1.23 ± 0.04 ** |
| VLDL cholesterol (mmol/l) | 0.35 ± 0.01 | 0.28 ± 0.01 ** | 0.47 ± 0.05 | 0.44 ± 0.22 |
| VLDL triacylglycerides (mmol/l) | 1.97 ± 0.08 | 1.15 ± 0.07 *** | 2.55 ± 0.22 | 0.89 ± 0.07 ** |
| total cholesterol (mmol/l) | 3.81 ± 0.15 | 3.73 ± 0.08 | 3.31 ± 0.18 | 4.08 ± 0.21 |
| ***clinical chemistry*** | ***(n = 31)*** | ***(n = 40)*** | ***(n = 26)*** | ***(n = 12)*** |
| ALAT (µkat/l) | 0.51 ± 0.29 | 0.50 ± 0.25 | 0.19 ± 0.07 | 0.22 ± 0.08 |
| ASAT (µkat/l) | 1.35 ± 0.65 | 1.27 ± 0.77 | 1.17 ± 0.66 | 0.95 ± 0.30 |
| AP (µkat/l) | 1.82 ± 0.67 | 1.53 ± 0.50 * | 1.52 ± 0.39 | 2.17 ± 0.49 ** |
| CHE (µkat/l) | 100.87 ± 19.76 | 95.39 ± 14.68 | 142.12 ± 19.81 | 133.83 ± 15.21 |
| CK (µkat/l) | 1.63 ± 1.44 | 1.31 ± 0.96 | 2.13 ± 2.18 | 0.90 ± 0.78 |
| urea (mmol/l) | 6.78 ± 1.46 | 7.66 ± 1.38 ** | 7.10 ± 1.54 | 6.63 ± 1.14 |
| calcium (mmol/l) | 2.78 ± 0.36 | 2.81 ± 0.19 | 2.76 ± 0.16 | 2.81 ± 0.15 |
| phosphate (mmol/l) | 3.43 ± 0.56 | 3.72 ± 0.55 ** | 3.19 ± 0.41 | 3.61 ± 0.62 |
| magnesium (mmol/l) | 1.45 ± 0.29 | 1.47 ± 0.25 | 1.40 ± 0.19 | 1.50 ± 0.20 |
| protein (g/l) | 59.25 ± 5.12 | 59.29 ± 4.95 | 59.85 ± 2.96 | 60.10 ± 3.15 |
| albumin (g/l) | 39.05 ± 4.65 | 38.30 ± 3.78 | 42.06 ± 2.47 | 43.05 ± 1.91 |
| triacylglycerides (mmol/l) | 3.05 ± 0.99 | 2.55 ± 0.84 ** | 2.80 ± 0.55 | 2.25 ± 0.58 * |
| cholesterol (mmol/l) | 4.31 ± 1.16 | 4.22 ± 1.14 | 3.13 ± 0.49 | 3.10 ± 0.33 |
| free T4 (pmol/l) | 35.32 ± 6.82 | 39.07 ± 3.34 * | 32.49 ± 6.04 | 31.85 ± 5.53 |
| urine osmolality | 2618 ± 669 | 2629 ± 691 | 2834 ± 744 | 2354 ± 532 |
