# Supplementary material for: Reduced Food Intake and Body Weight in Mice Deficient for the G Protein-Coupled Receptor GPR82
Source: PLoS One. 2011 Dec 28;6(12):e29400. doi: 10.1371/journal.pone.0029400 (PMC3247265; doi:10.1371/journal.pone.0029400)
Supplement: Table S6 — Phenotypical and laboratory screen for differences between WT and KO mice under Western-type diet. 15-week-old male and female WT and KO mice were kept under Western diet for 12 weeks and subjected to different analyses as mice under normal diet (see suppl. Table S3). Results are given as mean ± SD. *P<0.05, **P<0.01, ***P<0.001. (DOC) [file pone.0029400.s016.doc]

|  | ***male*** | | ***female*** | |
| --- | --- | --- | --- | --- |
| ***Parameter*** | ***WT*** | ***KO*** | ***WT*** | ***KO*** |
| ***body composition*** | ***(n = 10)*** | ***(n = 10)*** | ***(n = 9)*** | ***(n = 9)*** |
| body weight | 44.61 ± 1.62 | 39.32 ± 2.39 *** | 39.33 ± 2.11 | 38.55 ± 4.88 |
| fat (g) | 10.84 ± 0.82 | 8.72 ± 1.24 *** | 13.57 ± 1.16 | 12.36 ± 1.96 * |
| fat (% body weight) | 24.34 ± 1.24 | 22.09 ± 2.45 *** | 34.55 ± 2.46 | 32.50 ± 2.64 ** |
| lean (g) | 29.31 ± 1.12 | 26.43 ± 1.51 *** | 22.65 ± 1.24 | 22.06 ± 2.27 |
| lean (% body weight) | 65.87 ± 1.46 | 67.22 ± 2.20 ** | 57.70 ± 2.82 | 58.31 ± 3.48 |
| water (g) | 26.48 ± 0.98 | 23.92 ± 1.33 *** | 20.47 ± 1.11 | 19.93 ± 2.04 |
| water (% body weight) | 59.51 ± 1.35 | 60.85 ± 1.84 ** | 52.13 ± 2.56 | 52.70 ± 3.26 |
| ***oGTT*** | **(*n* = 19)** | **(*n* = 15)** | **(*n* = 21)** | **(*n* = 14)** |
| fasting blood glucose | 8.69 ± 1.41 | 7.64 ± 1.03 * | 7.26 ± 1.14 | 6.51 ± 1.16 * |
| *blood glucose after:* | | | | |
| 20 min | 16.26 ± 3.77 | 11.60 ± 2.08 *** | 11.54 ± 2.78 | 9.44 ± 1.67 ** |
| 40 min | 13.58 ± 4.45 | 8.64 ± 1.31 *** | 8.65 ± 1.48 | 7.81 ± 0.87 * |
| 60 min | 10.10 ± 2.70 | 8.07 ± 1.05 ** | 8.73 ± 1.61 | 7.94 ± 1.13 |
| 120 min | 9.23 ± 2.40 | 7.45 ± 0.88 ** | 7.49 ± 1.30 | 6.96 ± 1.20 |
| AUC | 1364 ± 304 | 1027 ± 118 *** | 1052 ± 164 | 936 ± 125 * |
| ***insulin concentration*** | **(*n* = 13)** | **(*n* = 5)** | **(*n* = 18)** | **(*n* = 5)** |
| insulin (ng/ml) | 1.39 ± 0.43 | 0.64 ± 0.24 *** | 1.44 ± 0.52 | 0.59 ± 0.07 *** |
| ***serum*** | **(*n* = 10)** | **(*n* = 10)** | **(*n* = 9)** | **(*n* = 9)** |
| HDL cholesterol (mmol/l) | 4.16 ± 0.14 | 4.84 ± 0.08 ** | 3.40 ± 0.19 | 3.20 ± 0.24 |
| HDL triaylglycerides (mmol/l) | 0.86 ± 0.07 | 0.99 ± 0.04 | 0.98 ± 0.04 | 0.96 ± 0.07 |
| LDL cholesterol (mmol/l) | 2.66 ± 0.15 | 2.28 ± 0.13 | 2.22 ± 0.24 | 2.02 ± 0.13 |
| LDL triacylglycerides (mmol/l) | 1.29 ± 0.13 | 1.33 ± 0.08 | 1.30 ± 0.06 | 1.30 ± 0.08 |
| VLDL cholesterol (mmol/l) | 0.96 ± 0.07 | 0.73 ± 0.05 * | 0.84 ± 0.09 | 0.95 ± 0.08 |
| VLDL triacylglycerides (mmol/l) | 2.97 ± 0.71 | 2.15 ± 0.22 * | 2.37 ± 0.17 | 1.83 ± 0.22 |
| total cholesterol (mmol/l) | 7.75 ± 0.28 | 8.01 ± 0.15 | 6.38 ± 0.38 | 6.11 ± 0.33 |
| ***clinical chemistry*** | | | | |
| ALAT (µkat/l) | 0.98 ± 0.50 | 0.80 ± 0.42 | 1.62 ± 1.2 | 1.45 ± 0.72 |
| ASAT (µkat/l) | 1.77 ± 0.89 | 1.19 ± 0.23 * | 2.65± 1.33 | 2.23 ± 0.76 |
| AP (µkat/l) | 1.55 ± 0.28 | 1.37 ± 0.30 | 2.02 ± 0.22 | 1.77 ± 0.24 |
| CHE (µkat/l) | 134.09 ± 13.51 | 106.85 ± 19.94 ** | 174.15 ± 29.22 | 166.27 ± 17.96 |
| GLDH (µkat/l) | 1.17 ± 1.53 | 0.23 ± 0.08 * | 1.13 ± 1.08 | 0.67 ± 0.49 |
| CK (µkat/l) | 0.63 ± 0.28 | 0.60 ± 0.24 | 0.51 ± 0.14 | 0.60 ± 0.20 |
| urea (mmol/l) | 7.02 ± 0.96 | 7.45 ± 1.17 | 7.25 ± 1.25 | 7.96 ± 3.36 |
| calcium (mmol/l) | 2.86 ± 0.15 | 2.66 ± 0.48 | 2.88 ± 0.24 | 2.73 ±0.35 |
| phosphate (mmol/l) | 3.76 ± 0.46 | 3.71 ± 0.63 | 3.23 ± 0.52 | 3.86 ± 1.14 |
| magnesium (mmol/l) | 1.66 ± 0.24 | 1.45 ± 0.24 | 1.77 ± 0.23 | 1.77 ± 0.38 |
| total protein (g/l) | 65.16 ± 4.67 | 59.45 ± 10.16 | 65.26 ± 3.57 | 66.71 ± 8.00 |
| albumin (g/l) | 38.22 ± 4.63 | 32.10 ± 8.04 * | 43.05 ± 3.02 | 43.00 ± 7.01 |
| triacylglycerides (mmol/l) | 3.62 ± 0.86 | 3.17 ± 0.97 | 2.51 ± 0.90 | 2.62 ± 0.84 |
| cholesterol (mmol/l) | 6.84 ± 0.51 | 6.01 ± 1.23 | 5.40 ± 0.91 | 5.20 ± 0.88 |
