# Supplementary material for: Reduced Food Intake and Body Weight in Mice Deficient for the G Protein-Coupled Receptor GPR82
Source: PLoS One. 2011 Dec 28;6(12):e29400. doi: 10.1371/journal.pone.0029400 (PMC3247265; doi:10.1371/journal.pone.0029400)
Supplement: Table S7 — Results of SHIRPA Protocol. Following the modified SHIRPA protocol [8], [9] WT and KO male mice were tested. Results are means of scores from 3 independent observers ± SD (http://phenome.jax.org/pub-cgi/phenome/mpdcgi?rtn=projects/docstatic&doc=Lake2/Lake2_Protocol). Open field and light/dark test: 3-month-old male mice were tested in the setup. Recording and analysis was performed automatically. The table shows main parameters as mean ± SD. *P<0.05; **P<0.01. (DOC) [file pone.0029400.s017.doc]

| ***SHIRPA protocol*** | ***WT (n = 8)*** | ***KO (n = 10)*** |
| --- | --- | --- |
| Body position | 3.25 ± 0.24 | 2.76 ± 0.47 |
| Spontaneous activity | 0.71 ± 0.28 | 0.67 ± 0.30 |
| Tremor | 0 | 0 |
| Palpebral closure | 0 | 0 |
| Coat appearance | 0.04 ± 0.12 | 0.06 ± 0.13 |
| Whiskers | 0 | 0 |
| Defecation | 2.88 ± 1.36 | 2.64 ± 2.38 |
| Transfer arousal | 1.13 ± 0.40 | 0.85 ± 0.38 |
| Gait | 0.67 | 0.73 ± 0.13 |
| Tail elevation | 0 | 0.03 ± 0.10 |
| Touch escape | 0.38 ± 0.45 | 0.18 ± 0.35 |
| Trunk curl | 0.08 ± 0.15 | 0.48 ± 0.46 |
| Limb grasping | 1 | 1 |
| Grip strength | 4 | 4 |
| Struggles when held by tail | 0 | 0 |
| Struggles when held by neck | 2.33 ± 0.50 | 2.52 ± 0.46 |
| Struggles when laid supine | 3 | 3 |
| Corneal Reflex | 0.13 ± 0.25 | 0.12 ± 0.31 |
| Toe pinch | 0.21 ± 0.35 | 0 |
| Pinna reflex | 0 | 0.03 ± 0.10 |
| Contact righting reflex | 0.04 ± 0.12 | 0.18 ± 0.27 |
| Wire maneuver | 1.50 ± 1.14 | 2.12 ± 1.40 |
| Negative geotaxis | 1 | 1.33 ± 0.75 |
| ***Open field test*** | | |
| Total activity (s) | 144.3 ± 37.8 | 119.0 ± 51.4 |
| Total activity (%) | 48.1 ± 12.6 | 39.7 ± 17.1 |
| Counts | 2019 ± 646 | 1337 ± 578 * |
| Velocity (cm s^-1^) | 20.8 ± 3.1 | 16.8 ± 1.8 ** |
| Total activity (%) | 48.1 ± 12.6 | 39.7 ± 17.1 |
| Distance (m) | 33.7 ± 10.2 | 24.0 ± 10.6 |
| Distance edges (m) | 32.6 ± 9.8 | 23.5 ± 10.0 |
| Distance center (m) | 1.1 ± 1.2 | 0.54 ± 0.80 |
| Distance corners (m) | 6.3 ± 5.0 | 6.5 ± 4.4 |
| Attendance center (%) | 3.1 ± 3.9 | 1.5 ± 2.7 |
| Attendance edges (%) | 97.0 ± 3.9 | 98.5 ± 2.7 |
| ***Light/dark test*** | | |
| Total activity (s) | 136.0 ± 56.6 | 104.3 ± 47.0 |
| Total activity (%) | 45.3 ± 18.9 | 34.8 ± 15.7 |
| Counts | 2003 ± 1072 | 1277 ± 605 |
| Velocity (cm s^-1^) | 19.9 ± 3.5 | 17.8 ± 2.3 |
| Attendance in the light part (s) | 244.1 ± 79.9 | 289.5 ± 30.2 |
| Distance in light part (m) | 21.9 ± 6.5 | 21.3 ± 11.3 |
| Activity in light part (s) | 98.1 ± 27.0 | 96.7 ± 49.3 |
| Attendance in the area between light and dark part (s) | 20.8 ± 14.8 | 7.2 ± 7.2 * |
| Distance area between light and dark part (m) | 2.6 ± 2.5 | 0.4 ± 0.5 |
| Activity in the area between light and dark part (s) | 11.8 ± 10.1 | 2.1 ± 3.1 * |
| Attendance in the dark part (s) | 56.9 ± 81.3 | 10.5 ± 30.2 |
| Distance in dark part (m) | 9.9 ± 14.1 | 1.3 ± 3.7 |
| Activity in dark part (s) | 38.7 ± 54.8 | 5.6 ± 16.4 |
| Total distance (m) | 31.6 ± 15.7 | 22.5 ± 11.3 |
