# Supplementary material for: Reduced Food Intake and Body Weight in Mice Deficient for the G Protein-Coupled Receptor GPR82
Source: PLoS One. 2011 Dec 28;6(12):e29400. doi: 10.1371/journal.pone.0029400 (PMC3247265; doi:10.1371/journal.pone.0029400)
Supplement: Table S8 — DTH test. WT and KO male animals were intradermally immunized by methylated BSA emulsified in CFA. At day 8 p.i. mBSA was injected intradermally into one hind paw and 0.9% saline into the other hind paw as a control. Paw swelling was measured at time points as indicated. For details see suppl. Methods. *P<0.05; **P<0.01; ***P<0.001 (DOC) [file pone.0029400.s018.doc]

|  | ***WT (n = 23)*** | | | ***KO (n = 20)*** | | |
| --- | --- | --- | --- | --- | --- | --- |
| ***time (h)*** | ***control paw*** | ***injected paw*** | ***swelling (%)*** | ***control paw*** | ***injected paw*** | ***swelling (%)*** |
| 0 | 1.46 ± 0.02 | 1.46 ± 0.02 | 100 | 1.46 ± 0.03 | 1.46 ± 0.03 | 100 |
| 12 | 1.46 ± 0.02 | 1.99 ± 0.15 | 135.24 ± 10.69 | 1.45 ± 0.02 | 1.92 ± 0.19 | 131.74 ± 13.76 |
| 24 | 1.44 ± 0.02 | 2.16 ± 0.18 | 150.02 ± 12.88 | 1.45 ± 0.02 | 2.02 ± 0.16 | 139.14 ± 11.31 ** |
| 36 | 1.45 ± 0.02 | 2.01 ± 0.14 | 138.78 ± 9.57 | 1.45 ± 0.02 | 1.87 ± 0.09 | 128.55 ± 5.92 *** |
| 48 | 1.45 ± 0.02 | 1.95 ± 0.13 | 134.95 ± 9.25 | 1.45 ± 0.02 | 1.87 ± 0.09 | 128.56 ± 6.24 * |
| 72 | 1.45 ± 0.02 | 1.85 ± 0.07 | 128.20 ± 5.04 | 1.46 ± 0.02 | 1.77 ± 0.08 | 121.54 ± 5.43 ** |
