# Supplementary material for: Reduced Food Intake and Body Weight in Mice Deficient for the G Protein-Coupled Receptor GPR82
Source: PLoS One. 2011 Dec 28;6(12):e29400. doi: 10.1371/journal.pone.0029400 (PMC3247265; doi:10.1371/journal.pone.0029400)
Supplement: Table S9 — Expression of selected genes involved in regulation of energy balance and food intake. Gene expression ratios are given as ΔCt values (mean ± SEM) with the quantitative (q) PCR sample size in parentheses. The mean Ct values of β2 microglobulin in stomach (WT: 20.3±0.2 vs. KO: 19.5±0.1), hypothalamus (WT: 20.8±0.1 vs. KO: 21.5±0.1), and liver (WT: 18.9±0.7 vs. KO: 20.0±0.2) were not significantly different between WT and KO. n.d. not determined, *P<0.05. (DOC) [file pone.0029400.s019.doc]

|  | ***hypothalamus*** | | ***liver*** | |
| --- | --- | --- | --- | --- |
| ***gene*** | ***WT (n = 9)*** | ***KO (n = 9)*** | ***WT (n = 5)*** | ***KO (n = 5)*** |
| POMC | 1.79 ± 0.34 | 2.25 ± 0.32 | n.d. | n.d. |
| Ghrelin receptor | 8.75 ± 0.54 | 8.93 ± 0.37 | n.d. | n.d. |
| NPY | -2.41 ± 0.39 | -1.55 ± 0.30 | n.d. | n.d. |
| AgRP | 0.52 ± 0.17 | 0.75 ± 0.38 | n.d. | n.d. |
| insulin receptor | n.d. | n.d. | 7.33 ± 0.48 | 7.65 ± 0.66 |
| leptin receptor | n.d. | n.d. | 9.36 ± 0.96 | 8.08 ± 0.35 * |
