# Supplementary material for: Reduced Food Intake and Body Weight in Mice Deficient for the G Protein-Coupled Receptor GPR82
Source: PLoS One. 2011 Dec 28;6(12):e29400. doi: 10.1371/journal.pone.0029400 (PMC3247265; doi:10.1371/journal.pone.0029400)
Supplement: Table S10 — Determination of the energy balance in metabolic cages. 20-week-old male mice were adapted to metabolic cages (Phenomaster, TSE Systems) and monitored over 24 h. Values are given as mean ± SD. *P<0.05; **P<0.01; ***P<0.001. (DOC) [file pone.0029400.s020.doc]

|  | ***day*** | | ***night*** | |
| --- | --- | --- | --- | --- |
|  | ***WT (n = 9)*** | ***KO (n = 8)*** | ***WT (n = 9)*** | ***KO (n = 8)*** |
| body weight (g) | 31.6 ± 0.9 | 28.5 ± 1.8 *** | 31.6 ± 0.9 | 28.5 ± 1.8 *** |
| VO_2_ (ml/h/kg) | 2318 ± 301 | 2535 ± 397 | 2945 ± 285 | 3136 ± 511 |
| VCO_2_ (ml/h/kg) | 1227 ± 578 | 1520 ± 788 | 1572 ± 703 | 1946 ± 1097 |
| respiratory rate | 0.83 ± 0.07 | 0.84 ± 0.05 | 0.87 ± 0.07 | 0.87 ± 0.06 |
| metabolic rate | 11.34 ± 1.49 | 12.36 ± 1.96 | 14.41 ± 1.55 | 15.39 ± 2.58 |
| locomotion |  |  |  |  |
| XY (counts) | 64.1 ± 32.6 | 47.8 ± 25.9 | 206.0 ± 124.7 | 127.5 ± 60.7 |
| Z (counts) | 11.5 ± 4.6 | 7.9 ± 3.9 | 33.3 ± 9.6 | 22.0 ± 7.0 * |
| distance (m) | 1359 ± 672 | 934 ± 672 | 1332 ± 677 | 903 ± 659 |
| wheel |  |  |  |  |
| max speed (m/s) | 0.89 ± 0.05 | 0.57 ± 0.34 * | 0.90 ± 0.05 | 0.80 ± 0.15 |
| aver. speed (m/s) | 0.30 ± 0.05 | 0.17 ± 0.10 ** | 0.25 ± 0.03 | 0.18 ± 0.07 * |
| max duration (s) | 369.2 ± 129.3 | 165.3 ± 113.9 ** | 410.5 ± 118.6 | 305.1 ± 181.2 |
