# Supplementary material for: Reduced Food Intake and Body Weight in Mice Deficient for the G Protein-Coupled Receptor GPR82
Source: PLoS One. 2011 Dec 28;6(12):e29400. doi: 10.1371/journal.pone.0029400 (PMC3247265; doi:10.1371/journal.pone.0029400)
Supplement: Table S11 — Phenotype-genotype correlation in the Sorbs cohort – Genotype frequency distribution. (A) ∼900 individuals (for details see Experimental Procedures) of the self-contained population of Sorbs were genotyped at 7 tagging SNP sites covering the genes for GPR34 and GPR82. Only homozygous female individuals were included in the association analysis (because males are hemizygous). Number of individuals homo-/hemizygous for the allele are given. (B) All effect directions (beta) in the association analysis were standardized to the minor allele and are shown with corresponding p-values. Only non-diabetic individuals and, for female individuals, only homozygotes (because males are hemizygous) were included. Associations with serum lipid parameters were assessed only in homo-/hemizygous individuals without lipid-lowering medication. Data are displayed for both genders. *P<0.05; **P<0.01. (DOC) [file pone.0029400.s021.doc]

A

|  | ***rs12851091*** | | ***rs913602*** | | ***rs1023064*** | | ***rs5918225*** | | ***rs5917441*** | | ***rs6609159*** | | ***rs4827286*** | |
| --- | --- | --- | --- | --- | --- | --- | --- | --- | --- | --- | --- | --- | --- | --- |
|  | C | G | C | T | C | T | A | T | A | G | C | T | C | T |
| n (total) | 10 | 867 | 523 | 120 | 239 | 431 | 620 | 107 | 832 | 24 | 258 | 407 | 457 | 213 |
| n (female) | 0 | 516 | 267 | 42 | 83 | 226 | 334 | 34 | 491 | 7 | 96 | 208 | 238 | 71 |
| n (male) | 10 | 351 | 256 | 78 | 156 | 205 | 286 | 73 | 341 | 17 | 162 | 199 | 219 | 142 |

B

| ***parameters*** | ***gender*** | ***rs12851091*** | ***rs913602*** | ***rs1023064*** | ***rs5918225*** | ***rs5917441*** | ***rs6609159*** | ***rs4827286*** |
| --- | --- | --- | --- | --- | --- | --- | --- | --- |
|  |  | beta / p | beta / p | beta / p | beta / p | beta / p | beta / p | beta / p |
| BMI [kg/m^2^] | female | - | -0.054 / 0.276 | 0.007 / 0.887 | -0.005 / 0.907 | 0.031 / 0.426 | -0.026 / 0.591 | 0.01 / 0.836 |
|  | male | **-0.098 / 0.039*** | 0.057 / 0.250 | **-0.118 / 0.013*** | 0.017 / 0.730 | 0.073 / 0.127 | **-0.122 / 0.01*** | **-0.126 / 0.008**** |
| body fat [%] | female | - | -0.083 / 0.119 | 0.052 / 0.315 | -0.009 / 0.854 | -0.007 / 0.875 | 0.016 / 0.763 | 0.053 / 0.302 |
|  | male | -0.075 / 0.136 | 0.077 / 0.139 | **-0.119 / 0.017*** | 0.043 / 0.395 | 0.034 / 0.492 | **-0.136 / 0.006**** | **-0.117 / 0.018*** |
| lean mass [%] | female | - | -0.023 / 0.692 | -0.003 / 0.965 | -0.010 / 0.841 | 0.066 / 0.143 | -0.016 / 0.775 | -0.022 / 0.698 |
|  | male | -0.079 / 0.136 | 0.063 / 0.254 | -0.075 / 0.157 | 0.053 / 0.317 | 0.039 / 0.467 | -0.069 / 0.19 | **-0,107 / 0.043*** |
| glucose [mmol/l] (0 min) | female | - | 0.004 / 0.941 | -0.038 / 0.424 | -0.039 / 0.372 | 0.022 / 0.556 | -0.008 / 0.871 | 0.007 / 0.88 |
|  | male | -0.024 / 0.618 | -0.069 / 0.164 | 0.016 / 0.734 | -0.053 / 0.264 | -0.039 / 0.412 | 0.011 / 0.811 | 0.008 / 0.864 |
| glucose [mmol/l] (30 min) | female | - | -0.002 / 0.970 | 0.005 / 0.930 | -0.020 / 0.670 | 0.027 / 0.515 | -0.013 / 0.802 | 0.022 / 0.673 |
|  | male | 0.077 / 0.132 | -0.027 / 0.614 | 0.016 / 0.747 | -0.060 / 0.239 | 0 / 0.998 | 0.026 / 0.610 | -0.008 / 0.873 |
| glucose [mmol/l] (120 min) | female | - | 0.261 / 0.060 | **-0.106 / 0.043*** | 0.044 / 0.351 | 0.056 / 0.171 | **-0.120 / 0.022*** | -0.069 / 0.182 |
|  | male | 0.038 / 0.447 | -0.025 / 0.632 | 0.058 / 0.239 | -0.017 / 0.724 | -0.064 / 0.190 | **0.096 / 0.050*** | **0.110 / 0.026*** |
| triacylgly-cerides [mmol/l] | female | - | -0.024 / 0.646 | 0.011 / 0.830 | 0.019 / 0.691 | -0.019 / 0.653 | 0.017 / 0.744 | 0.022 / 0.680 |
|  | male | -0.043 / 0.370 | 0.014 / 0.784 | 0.012 / 0.796 | 0.015 / 0.75 | -0.020 / 0.686 | 0.027 / 0.566 | 0.032 / 0.502 |
| cholesterol [mmol/l] | female | - | -0.057 / 0.254 | 0.041 / 0.418 | 0.020 / 0.666 | -0.007 / 0.851 | 0.042 / 0.415 | 0.022 / 0.666 |
|  | male | -0.045 / 0.355 | 0.012 / 0.814 | -0.035 / 0.470 | 0.020 / 0.679 | -0.012 / 0.812 | -0.022 / 0.657 | 0.002 / 0.965 |
