# Supplementary material for: Reduced Food Intake and Body Weight in Mice Deficient for the G Protein-Coupled Receptor GPR82
Source: PLoS One. 2011 Dec 28;6(12):e29400. doi: 10.1371/journal.pone.0029400 (PMC3247265; doi:10.1371/journal.pone.0029400)
Supplement: Table S12 — BMI-genotype correlation at tagging SNP rs6609159 and rs4827286. (A, B) ∼530 individuals of a cohort from Leipzig [10] were genotyped at two tagging SNP sites (rs6609159, rs4827286) that showed strongest association with BMI in the Sorbs cohort (see above). (C, D) ∼1900 individuals of the Leipzig Heart Study [11] were genotyped at two tagging SNP sites (rs6609159, rs4827286). (E, F) Meta analysis of the two additional cohorts together with the Sorbs cohort (∼3330 individuals) revealed significant correlations between genotypes and BMI. For female individuals only homozygous individuals were included in the association analysis (because males are hemizygous). Numbers of individuals homo-/hemizygous for the allele are given in A, C, E). All effect directions (beta) in the association analysis were standardized to the minor allele and are shown with corresponding p-values (B, D, F). *P<0.05; **P<0.01. (DOC) [file pone.0029400.s022.doc]

| **A** | **B** |
| --- | --- |
| |  | ***rs6609159*** | | ***rs4827286*** | | | --- | --- | --- | --- | --- | | C | T | C | T | | n (total) | 138 | 175 | 224 | 105 | | n (female) | 84 | 120 | 161 | 60 | | n (male) | 54 | 55 | 63 | 45 | | | ***parameter*** | ***gender*** | ***rs6609159*** | ***rs4827286*** | | --- | --- | --- | --- | |  |  | beta / p | beta / p | | BMI [kg/m^2^] | total | -0.026 / 0.620 | 0.005 / 0.921 | | female | 0.005 / 0.944 | 0.037 / 0.552 | | male | -0.096 / 0.284 | -0.069 / 0.441 | |
|  |  |
| **C** | **D** |
| |  | ***rs6609159*** | | ***rs4827286*** | | | --- | --- | --- | --- | --- | | C | T | C | T | | n (total) | 668 | 867 | 1031 | 525 | | n (female) | 136 | 209 | 282 | 85 | | n (male) | 532 | 658 | 749 | 440 | | | ***parameter*** | ***gender*** | ***rs6609159*** | ***rs4827286*** | | --- | --- | --- | --- | |  |  | beta / p | beta / p | | BMI [kg/m^2^] | total | -0.019 / 0.463 | -0.037 / 0.149 | | female | 0.030 / 0.582 | 0.020 / 0.700 | | male | -0.035 / 0.226 | -0.054 / 0.062 | |
|  |  |
| **E** | **F** |
| |  | ***rs6609159*** | | ***rs4827286*** | | | --- | --- | --- | --- | --- | | C | T | C | T | | n (total) | 1064 | 1449 | 1712 | 843 | | n (female) | 316 | 537 | 681 | 216 | | n (male) | 748 | 912 | 1031 | 627 | | | ***parameter*** | ***gender*** | ***rs6609159*** | ***rs4827286*** | | --- | --- | --- | --- | |  |  | beta / p | beta / p | | BMI [kg/m^2^] | total | -0.018 / 0.351 | -0.029 / 0.132 | | female | 0.021 / 0.503 | 0.035 / 0.263 | | male | **-0.047 / 0.050*** | **-0.069 / 0.004**** | |
