# Supplementary material for: Reduced Food Intake and Body Weight in Mice Deficient for the G Protein-Coupled Receptor GPR82
Source: PLoS One. 2011 Dec 28;6(12):e29400. doi: 10.1371/journal.pone.0029400 (PMC3247265; doi:10.1371/journal.pone.0029400)
Supplement: Methods S1 — Supplementary Methods. (DOC) [file pone.0029400.s023.doc]

## Supplemental Material

**Reduced Food Intake and Body Weight in mice deficent for the G Protein-coupled Receptor GPR82**

**Kathrin M. Y. Engel^1+^, Kristin Schröck^1+^, Daniel Teupser^2^, Lesca Miriam Holdt^2^, Anke Tönjes^3^, Matthias Kern^3^, Kerstin Dietrich^4^, Peter Kovacs^4^, Ute Krügel^5^, Holger A. Scheidt^6^, Jürgen Schiller^6^, Daniel Huster^6^, Gudrun A. Brockmann^7^, Martin Augustin^8^, Joachim Thiery^2^, Matthias Blüher^3^, Michael Stumvoll^3^, Torsten Schöneberg^1#^, and Angela Schulz^1^**

^1^Molecular Biochemistry, Institute of Biochemistry, Medical Faculty, University of Leipzig, Leipzig, Germany, ^2^Institute of Laboratory Medicine, Clinical Chemistry and Molecular Diagnostics, Medical Faculty, University of Leipzig, Leipzig, Germany, ^3^Department of Internal Medicine, Medical Faculty, University of Leipzig, Leipzig, Germany, ^4^Interdisciplinary Centre for Clinical Research, Medical Faculty, University of Leipzig, Leipzig, Germany, ^5^Rudolf Boehm Institute of Pharmacology and Toxicology, Medical Faculty, University of Leipzig, Leipzig, Germany, ^6^Institute of Medical Physics and Biophysics, Medical Faculty, University of Leipzig, Leipzig, Germany, ^7^Institute of Animal Sciences, Humboldt-Universität zu Berlin, Berlin, Germany, ^8^Ingenium Pharmaceuticals AG, Martinsried, Germany, now Millipore Corporation, St. Charles, Missouri, USA

^#^To whom correspondence should be addressed: Torsten Schöneberg, Institute of Biochemistry, Molecular Biochemistry, Medical Faculty, University of Leipzig, Leipzig, Germany, Phone: +49-341-9722-151, Fax: +49-341-9722-159, E-mail: schoberg@medizin.uni-leipzig.de

## SUPPLEMENTAL Methods

*Generation of GPR82-KO mice* - Mice were generated by homologous recombination. The target vector contained GPR82-flanking genomic sequence and an EMCV-internal ribosome entry site/β-galactosidase/neomycin (IRES-lacZ-neo)-fusion gene replacing most of the coding exon of GPR82 (insertion between codon 1 and 254) (*suppl. Figure S2A*). Neomycin-resistant ES cells were screened for homologous recombination. Positive ES cell clones were injected into blastocysts. Chimeric offspring were crossed into a C3HeB/FeJ background. Correct homologous recombination and correctness of the inserted cassette were verified by sequencing an 8.7-kb PCR product spanning target vector internal and external sequences. This PCR product was amplified from genomic KO mouse DNA using the Expand Long Template PCR System (Roche Applied Science, Mannheim, Germany) and the primers: 5’-TCA CAG GGA AGA CTT AAA AGG AAA CAC CAG AG-3’; 5’-AGA ACT CTG GGC ATT TCA GAC AGT CTC CTA CT-3’. The cycling conditions were 2 min 94°C, 10 x (10 s 94°C, 30 s 60°C, 13 min 68°C), 30 x (15 s 94°C, 30 s 60°C, 13 min 68°C) and finally 7 min 68°C. DNA from a WT mouse was used as control resulting in a 5-kb fragment.

*Genotyping of animals -* Routine genotyping of mice was performed by multiplex PCR (*suppl. Figure S2*). The genomic sequence was amplified using a touchdown-PCR protocol with the following conditions: 95°C for 30 s, 61°C for 30 s and 72°C for 30 s in the first two cycles. Then, the annealing temperature decreased 2°C every two cycles. After 6 cycles the conditions changed to 95°C for 30 s, 61°C for 30 s and 72°C for 30 s for another 28 cycles. The KO allele appears as a 272-bp band on an agarose gel, while the WT allele shows a 232-bp band (*suppl. Figure S2B*).

*Morphology and histology* - Weights of brain, heart, lung, liver, left and right kidney, spleen, (testes and epididymis only in males) were recorded for 31 WT and 24 KO male mice as well as for 20 WT and 31 KO female mice. For histological analysis organs were embedded in Tissue Tek (Sakura, Torrance, CA, USA) and kept at -80°C until cryosection. Tissue sections (20 µm) stained with nuclear fast red and H&E were microscopically investigated.

*Blood chemistry* - Animals were sacrificed by CO_2_ and cervical dislocation and blood was drawn from the *Vena cava inferior*. Metabolites, enzymes and hormones were analyzed in serum or, where appropriate, whole blood, according to the guidelines of the German Society of Clinical Chemistry and Laboratory Medicine, using a Hitachi PPEModular analyzer (Roche Diagnostics, Mannheim, Germany), a FreeStyle Lite instrument (Abbott Diabetes Care Ltd., Oxon, UK) and for AA/AC-Screen an electrospray ionization tandem mass spectrometer (ESI-MS/MS, API 2000, Applied Biosystems, Darmstadt, Germany) [1,2]. Ultra centrifugation was used to separate HDL, LDL and VLDL fractions.

*Hot plate test* *-* To confirm the hot plate test results from the initial SHIRPA screen (*suppl. Table S6*), this test was repeated at 52°C with 31 WT and 23 KO male mice as well as 19 WT and 19 KO female mice using a Hot Plate 602001 (TSE Technical & Scientific Equipment GmbH, Bad Homburg, Germany). The time it took the mouse to react to the heat stimulus was recorded. As reaction shaking or licking of one of the hind paws as well as jumping off the analgesia meter was used.

*Magnetic resonance measurements* – To measure the whole body fat composition mice (12 to 14 weeks of age) were examined by quantitative magnetic resonance (QMR) analysis using an echoMRI system (whole body composition analyzer, Echo Medical Systems, Houston, TX, USA). In a challenging experiment, mice were kept under a Western Type Diet EF R/M modified after TD88137 containing 21.2% fat and 32.8% carbohydrates (Ssniff, Soest, Germany) for 12 weeks and QMR analysis was performed. Additionally, all mice were analyzed in oGTT and serum lipids.

*Delayed Type Hypersensitivity (DTH) response* - Evaluation of DTH responses to methylated BSA (mBSA; Sigma) was performed according to Nakae et al. [3]. At day one mice were immunized intradermally with 200 µl of 2 mg/ml mBSA emulsified with CFA. At day 8 mice were injected intradermally with 20 µl of 5 mg/ml mBSA in 0.9% NaCl solution into one footpad and an equal volume of solely 0.9% NaCl solution into the other footpad as control. Footpad swelling was measured every 12 h over a total time period of 72 h using a millimeter screw (Kroeplin GmbH, Schlüchtern, Germany). DTH reaction was determined as difference between thickness of mBSA-injected footpad and thickness of NaCl solution-injected footpad.

*oGTT and ipGTT measurements* – Oral (oGTT) and intraperitoneal (ipGTT) glucose tolerance tests were performed. For oGTT 80 mg glucose were applied orally after a starving period over night. For ipGTT 2 mg glucose per g body weight were injected intraperitoneally into starved mice. Blood glucose concentrations were measured from tail vein blood before and 15, 30, 60 and 120 min after glucose application using a FreeStyle Lite instrument (Abbott Diabetes Care Ltd.).

*Insulin tolerance test (ITT)* – Changes in blood glucose levels in response to insulin were determined in an ITT. In the morning 0.75 U insulin (Actrapid U40) per kg body weight were injected i.p.. Blood glucose concentrations were measured from tail vein blood before and 15, 30 and 60 min after insulin application using a FreeStyle Lite instrument (Abbott Diabetes Care Ltd.).

*NMR measurements* – For NMR measurements the tissue samples were transferred into 4 mm HRMAS rotors with spherical Kel-F inserts. ^1^H HRMAS NMR experiments were carried out on Bruker NMR spectrometers with resonance frequencies of either 749.7 or 600.1 MHz. A 4 mm double-resonance MAS probe was used for the measurements applying excitation pulses of 4 to 12 µs in length. The MAS frequency was 9 kHz and the temperature was set to 303 K. The influence of the relaxation delay on peak intensities was tested by applying relaxation delays between 15 and 2.5 s. Since only minor differences were detected, a relaxation delay of 2.5 s was finally applied for all measurements. For peak integration, the Bruker TOPSPIN software package was used.

*MALDI-TOF measurements* – The triacylglyceride composition of brown, subcutaneous and perigonadal adipose tissue was determined by MALDI-TOF mass spectrometry. Lipids were eluted from 10 mg fat tissue by a mixture of chloroform, methanol and 0.9% NaCl (1/1/1, v/v/v). A 0.5 mol/l 2,5-dihydroxybenzoic acid (DHB) solution in methanol was used as matrix in all cases. All samples were premixed with the matrix prior to application onto the MALDI target. All MALDI-TOF mass spectra (positive polarity only) were recorded on a Bruker Autoflex mass spectrometer (Bruker Daltonics, Bremen, Germany). This system utilizes a pulsed nitrogen laser, emitting at 337 nm. One hundred single laser shots were averaged for each mass spectrum.

*Motor activity and feeding measurements* *-* The spontaneous motor activity of single-housed WT and KO mice was monitored over 3 weeks in TSE InfraMot (TSE Systems). Sensors, mounted on the lid of each home cage, registered the motor activity (including brief movements and rearing) of the subjects by sensing the body-heat image, i.e. infrared radiation, and its spatial displacement over time. The animals had free access to food and water. Food was delivered by special baskets projecting into the cage and connected to weight sensors of a LabMaster system (TSE Systems). The removal of food was monitored continuously by amount and time and simultaneously to the motor activity in 1 h intervals.

*Respiratory and metabolic rates measurements* *-* The respiratory and metabolic rates of single-housed WT and KO mice were continuously monitored over 24 h in a Phenomaster (TSE Systems).

## references

1. Ceglarek U, Muller P, Stach B, Buhrdel P, Thiery J, et al. (2002) Validation of the phenylalanine/tyrosine ratio determined by tandem mass spectrometry: sensitive newborn screening for phenylketonuria. Clin Chem Lab Med 40: 693-697.

2. Mueller P, Schulze A, Schindler I, Ethofer T, Buehrdel P, et al. (2003) Validation of an ESI-MS/MS screening method for acylcarnitine profiling in urine specimens of neonates, children, adolescents and adults. Clin Chim Acta 327: 47-57.

3. Nakae S, Komiyama Y, Nambu A, Sudo K, Iwase M, et al. (2002) Antigen-Specific T Cell Sensitization Is Impaired in IL-17-Deficient Mice, Causing Suppression of Allergic Cellular and Humoral Responses. 17: 375-387.

4. Huster D, Arnold K, Gawrisch K (1998) Influence of docosahexaenoic acid and cholesterol on lateral lipid organization in phospholipid mixtures. Biochemistry 37: 17299-17308.

5. Teuber K, Schiller J, Fuchs B, Karas M, Jaskolla TW (2010) Significant sensitivity improvements by matrix optimization: a MALDI-TOF mass spectrometric study of lipids from hen egg yolk. Chem Phys Lipids 163: 552-560.

6. Barrett JC, Fry B, Maller J, Daly MJ (2005) Haploview: analysis and visualization of LD and haplotype maps. Bioinformatics 21: 263-265.

7. Livak KJ, Schmittgen TD (2001) Analysis of Relative Gene Expression Data Using Real-Time Quantitative PCR and the 2-[Delta][Delta]CT Method. Methods 25: 402-408.

8. Rogers DC, Fisher EMC, Brown SDM, Peters J, Hunter AJ, et al. (1997) Behavioral and functional analysis of mouse phenotype: SHIRPA, a proposed protocol for comprehensive phenotype assessment. Mammalian Genome 8: 711-713.

9. Rogers DC, Peters J, Martin JE, Ball S, Nicholson SJ, et al. (2001) SHIRPA, a protocol for behavioral assessment: validation for longitudinal study of neurological dysfunction in mice. Neurosci Lett 306: 89-92.

10. Bottcher Y, Unbehauen H, Kloting N, Ruschke K, Korner A, et al. (2009) Adipose tissue expression and genetic variants of the bone morphogenetic protein receptor 1A gene (BMPR1A) are associated with human obesity. Diabetes 58: 2119-2128.

11. Holdt LM, Beutner F, Scholz M, Gielen S, Gabel G, et al. (2010) ANRIL expression is associated with atherosclerosis risk at chromosome 9p21. Arterioscler Thromb Vasc Biol 30: 620-627.
